# Regulation of social hierarchy learning by serotonin transporter availability

**DOI:** 10.1038/s41386-022-01378-2

**Published:** 2022-08-09

**Authors:** Remi Janet, Romain Ligneul, Annabel B. Losecaat-Vermeer, Remi Philippe, Gabriele Bellucci, Edmund Derrington, Soyoung Q. Park, Jean-Claude Dreher

**Affiliations:** 1 CNRS-Institut de Sciences Cognitives Marc Jeannerod, UMR5229, Neuroeconomics, reward, and decision making laboratory, Bron, France; 2 Champalimaud Neuroscience Program, Champalimaud Center for the Unknown, Lisbon, Portugal; 3 Neuropsychopharmacology and Biopsychology Unit, Department of Cognition, Emotion, and Methods in Psychology, Faculty of Psychology, University of Vienna, Vienna, Austria; 4 Charité-Universitätsmedizin Berlin, Corporate Member of Freie Universität Berlin, Humboldt-Universität zu Berlin, and Berlin Institute of Health, Neuroscience Research Center, 10117 Berlin, Germany; 5 Department of Computational Neuroscience, Max Planck Institute for Biological Cybernetics, Tübingen, Germany; 6 Department of Decision Neuroscience and Nutrition, German Institute of Human Nutrition (DIfE), Potsdam-Rehbrücke, Nuthetal, Germany

**Keywords:** Cognitive neuroscience, Learning and memory, Human behaviour

## Abstract

Learning one’s status in a group is a fundamental process in building social hierarchies. Although animal studies suggest that serotonin (5-HT) signaling modulates learning social hierarchies, direct evidence in humans is lacking. Here we determined the relationship between serotonin transporter (SERT) availability and brain systems engaged in learning social ranks combining computational approaches with simultaneous PET-fMRI acquisition in healthy males. We also investigated the link between SERT availability and brain activity in a non-social control condition involving learning the payoffs of slot machines. Learning social ranks was modulated by the dorsal raphe nucleus (DRN) 5-HT function. BOLD ventral striatal response, tracking the rank of opponents, decreased with DRN SERT levels. Moreover, this link was specific to the social learning task. These findings demonstrate that 5-HT plays an influence on the computations required to learn social ranks.

## Introduction

Competitive interactions shape the social hierarchy. Animals often need to fight to have access to resources (e.g. food, sexual partners) and the outcomes of these dyadic competitive interactions determine the social hierarchy in the group. In practice, a dominance relationship is considered as established when one individual repeatedly avoids competitive conflict with another [1]. Learning one’s social status during competitive dyadic interactions in a group is crucial to adapt behavior and avoid harmful social defeats [2, 3]. In animals, serotonin (5-HT) has been tightly coupled with social rewards and the establishment of social hierarchies [4–6]. For example, in groups of vervet monkeys, enhancement or suppression of serotonin signaling can induce dominance or subordination respectively [7]. Higher status ranked monkeys have more gray matter in the dorsal raphe nucleus (DRN) where serotoninergic neurons are located [8, 9]. Although indirect evidence from preclinical, pharmacological, and clinical studies suggests an association between the serotoninergic transporter from the DRN and neural responses related to learning social dominance hierarchies [10], there has been no demonstration of such a link in humans.

Here, using reinforcement-learning (RL) computational modeling and simultaneous PET-fMRI acquisition in the same individuals, we investigated the link between brain activity during social dominance hierarchy learning and a measure of serotoninergic function provided by serotonin transporter (SERT) availability. Participants were led to believe that they were interacting with opponents and had to learn their skills in a competitive game. On each trial, participants had to choose between two opponents among three, before competing against the chosen opponent. Although the exact role of 5-HT in RL has remained elusive, several studies have associated 5-HT signaling with diverse rewards and punishments [11, 12], including social rewards [5, 13]. DRN 5-HT neurons respond to both rewards and punishments, with modulations of tonic activity by context and phasic responses during reinforcer delivery, even when they were predicted [5, 14]. A recent optogenetic study reported that the learning rate may be under modulation of DRN 5-HT neurons [15]. This learning rate determines the number of trials over which reward histories are integrated to assess the value of actions that have been taken.

Based on these animal experiments, we reasoned that victories during social hierarchy learning through competitions may act as social rewards and that 5-HT levels may modulate the expected value of social rewards accumulated over all consecutive competitions, to represent the opponent’s social dominance status (SDS). This variable, reflecting the expected value (*Q*-value), is commonly used in model-free RL to learn the (Q)uality of actions to take in a given state. Q-learning determines an optimal action-selection policy by maximizing the expected value of total (discounted) rewards. Based on a previous fMRI study, we hypothesized that tracking the social dominance status (SDS) and the social prediction error (SPE) will engage the bilateral ventral striatum (VS) and the anterior medial prefrontal cortex (amPFC) while participants learned the relative ranks of their opponents [16]. We also hypothesized that inter-individual variations in SERT levels in the DRN would covary with the bilateral ventral striatum which encodes the social dominance status of the opponents because SERT is primarily distributed in the DRN and subcortical regions [17]. Confirming our hypothesis, we observed that individuals with higher SERT in the DRN showed reduced VS activity associated with the computation of the social dominance status of the opponent while learning social ranks. In contrast, no such relationship was observed during a similar non-social task. Moreover, the strength of the relationship between BOLD striatal signal and SERT levels in the DRN differed between the social and non-social learning conditions. These findings indicate a key role of 5-HT signaling in modulating the learning of social dominance relationships in humans.

## Methods

### Participants

Thirty-two healthy volunteers (only males and mean age (M)23.4 ± (SD)2.9) were recruited through a mailing list from the University Claude Bernard Lyon-1. Volunteers were screened by a physician for general MRI counter-indications and inclusion criteria. Two participants were excluded from the analysis, one because he expressed serious doubts about the cover story (cf. supplementary data) and the other because of a technical issue with the PET acquisition. Participants gave their written consent and received monetary compensation for the completion of the study. This study was approved by the Medical Ethics Committee (CPP Sud-Est IV, ID RCB: 2016-A01588-43).

### Experimental design

#### Social dominance hierarchy learning task

**Fig. 1:**
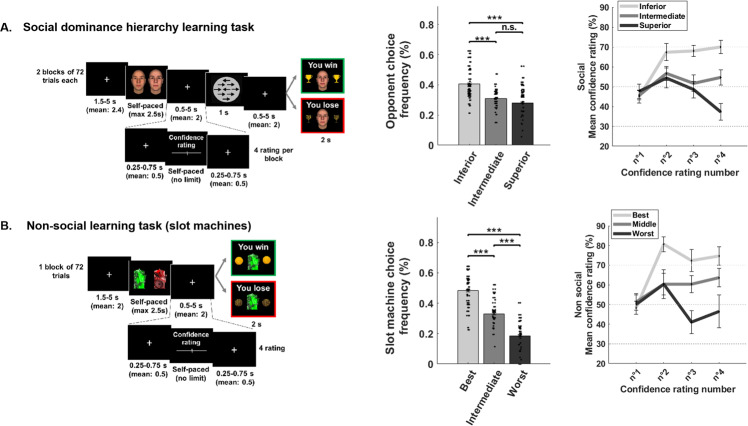
Tasks and behavioral results. **A** Social Dominance Hierarchy Learning task (left). Participants were led to believe they were competing against one of three real opponents. Unbeknownst to them, the probability they would win was predefined at *P* = 28%, 50%, and 72% for the superior, intermediate, and inferior opponents, respectively. In any one trial participants chose which one of two opponents they preferred to “compete” against. After competing in a perceptual decision-making task (circle with arrows), the outcome of the competition was delivered. For some trials (four per opponent), participants rated how confident they were of winning against the selected opponent (an example is shown in the bottom part of the panel). Note that the faces are derived from Todorov’s “25 Maximally Distinct Identities, Dominance” set. Middle: bar graphs represent the frequency with which they selected each opponent. Participants preferred to select the opponent against whom they had more chance of winning. Right: illustration of confidence rating through the social learning task. It is divided into the three reward probabilities (see Table S4 for more details on confidence rating). **B** Reinforcement Learning paradigm. Similar to the Social hierarchy learning task, participants chose which one of two slot machines among 3 they preferred to bet on (unknown winning probabilities: *P* = 28%, 50%, and 72% for the worst, intermediate and best chance to win, respectively). This was followed by an outcome phase in which they were informed if they had won or lost. In some trials, participants estimated how confident they were of winning on the selected slot machine (see bottom part of the panel). Middle: bar graphs represent the frequency that each slot machine was chosen. Right: illustration of the confidence rating through the non-social learning task. It is divided into the three reward probabilities. ***p < 0.001.

In the PET-fMRI social dominance hierarchy learning task, participants were led to believe that they were competing against three other participants anonymously connected online (Fig. 1A). For each trial participants first had to select against which opponent, between the two presented on the screen, they were going to play against. Then they had to play a round of the competitive perceptual decision-making task. Unbeknownst to the subjects, outcomes were manipulated to produce three different probabilities of winning called the reward probability (28%, 50%, or 72% of victories), depending on which of the 3 possible opponents they had chosen to play. After a fixation cross following the perceptual decision-making, subjects received feedback concerning the outcome of the competition, which was externally determined according to the defined probability. In some trials (one at the beginning, one at the end, and two in the middle of the task, resulting in four ratings according to the participant choice), participants were asked to indicate their confidence level with respect to their probability of winning against the selected opponent (Fig. 1A). Note that the opponents’ faces are derived from Todorov’s “25 Maximally Distinct Identities, Dominance” set [18].

Importantly, in the social dominance hierarchy learning task, winning or losing against opponents was not associated with monetary incentives but only with social victories or social defeats. Subjects played 2 games of 72 trials (24 trials per pair of opponents) for each run of the task. More detailed explanations could be found in the supplementary section covert story.

#### Non-social learning task (slot machines)

The non-social learning task was formally similar to the social hierarchy learning task, except that participants were not led to believe that they were in social interaction and thus did not have to compete against opponents. Instead, participants were told to choose between 2 slot machines from a group of three possible slots machines on each trial. Each slot machine had a defined probability of allowing the participants to win (28%, 50%, or 72%). After a fixation cross, participants received feedback about whether they won or lost based on the reward probability. As in the social hierarchy learning task, in some trials, participants were asked to indicate their confidence level with respect to the probability of winning with the slot machine they had selected on that trial (Fig. 1B).

Participants performed both the social and the non-social tasks while being scanned.

#### Computation of the competitive index

The competitive index was based on the opponents presented on the screen. For each trial, a competitive value of 1 was assigned to the stronger opponent, based on the predefined strength, and a competitive value of 0 for the weaker. Then, the proportion of competitive choices was computed by summing the competitive value in each trial divided by the number of trials played by participants. A value close to 1 represents a highly competitive index, whereas a value close to 0 reflects a non-competitive index. As competitive indexes were not normally distributed, we performed a Scheirer–Ray–Hare implemented in MATLAB (version 9.5.0.94, R2018b, Natick, Massachusetts: The MathWorks Inc). Mann–Whitney *post-hoc* tests were then performed on each bin to compare the competitive index of the two groups.

### PET and fMRI preprocessing

**Fig. 2:**
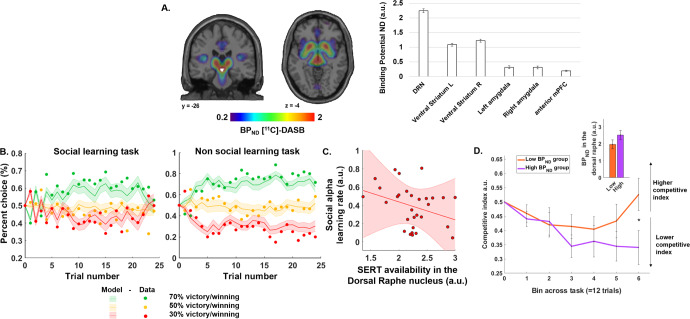
Binding potential in the dorsal raphe nucleus modulates social learning and competitive behavior. **A** Statistical map of the average BP_ND_ revealed a large distribution in the striatum and DRN. The white shape represents the DRN ROI. The bar graph represents the mean binding potential extracted in the DRN, ventral striatum left and right, amygdala left and right, and anterior PFC for illustration purposes. All extractions were performed using the ROI defined with the AAL3. **B** Left. Participants’ choice frequency during the social dominance learning task (dots) when facing the Inferior (green), Intermediate (orange), and Superior (red) opponents and model choice probability estimated by the RL algorithm. Right. Same illustration for the RL task. task. **C** Negative correlation between the BP_ND_ DRN, and participants’ learning rate in the social task. No correlation was observed between BP_ND,_ DRN and learning rate in the non-social task. **D** Competitive behavior related to the SERT level in the DRN. Competitive choices in the High and Low BP_ND_ groups. Individuals with lower BP_ND,_ in the DRN tended to increase their competitive choices, i.e. they chose to play against the stronger of the two opponents, in later trials. Interaction between trial bins and group (Low vs high BP_ND_ resulting from the median split) (*F*(_1,5_) = 4.41, *p* = 0.037). *Post-hoc* tests conducted on the last bin revealed that the high BP_ND_ group made less competitive choices in the last bin of the task (Median rank = 18.87) compared to the low BP_ND_ group (Median rank = 12.13) (*p* = 0.036). The bar graphs show a between-groups difference in BP_ND_ level in the DRN based on a median split of individuals. Errors bars represent SEM. ***p < 0.001. BP_N_ non-displaceable binding potential, DRN dorsal raphe nucleus, RL reinforcement learning.

PET and MRI acquisition performed simultaneously on a Siemens Biograph mMR. A detailed explanation of the preprocessing performed on the PET and fMRI data is provided in the supplementary data, section PET and fMRI preprocessing. For illustrative purposes, a brain statistical map resulting from the preprocessing is displayed in Fig. 2A. An individual gray matter mask was applied to the statistical maps. All statistical maps and extraction were performed after having applied a gray matter mask to each individual’s scan. For all GLM, participant motion parameters, the translations, and rotations framewise estimates were added into the first level GLM specification as regressors of non-interest to control for any effect related to motion.

### fMRI analyses

#### Encoding of the social dominance status of the selected opponent

A first GLM (GLM1), allowed us to investigate the brain regions encoding the social dominance status of the opponent (SDS(*t* + 1)) (i.e. choice-value) computed by summing αSPE(*t*) and SDS(*t*). This choice-value represents the dominance status of the opponent selected for that trial which will be updated based on the results of the trial and serves to guide future decisions. GLM1 included one categorical boxcar regressor of interest representing the outcomes phase (victory or defeat) with a fixed duration of 2 s. SDS(*t* + 1) was added as a parametric regressor to this categorial onset. This parametric modulator was previously normalized using the Fisher z-score transformation. In addition to this regressor of interest, GLM1 also included three others regressors. The first denoted the choice stage, which was parametrically modulated by the difficulty of the choice (computed as 1 − |choice probability – 0.5|) and the decision reaction time, previously normalized using the Fisher *z*-score transformation. This regressor was modeled as a boxcar function with the duration of the choice. The second regressor represented the confidence rating, modeled as a boxcar function with the duration of the rating. The last regressors represented the perceptual decision competition and were parametrically modulated by the accuracy and the reaction time. It was modeled as a boxcar function with the duration of the RT to respond to the perceptual decision competition. In addition, two regressors of no-interest were included and denoted both the missed choice and the missed competition as separate regressors.

#### Dissociating brain representations of the social dominance status of the opponent and prediction error

To investigate the relative variance explained by both the social prediction error SPE(*t*) and the SDS(*t*) for updating the new social dominance status of the opponent SDS(*t* + 1), we created GLM2. GLM2 is similar to GLM1 except that it includes two parametric regressors to the categorial boxcar regressors of interest, representing the outcome phase with a duration of 2 s. The two parametric regressors added were the social dominance status of the opponent SDS(*t*) and the social prediction errors, SPE(t), computed by the reinforcement-learning algorithm. The orthogonalization procedure was disabled to give equal “weight” to each of the parametric modulators, (SDS(*t*) and SPE(*t*)), related to the outcome phase, and to let them compete to explain the variance. These parametric modulators were previously normalized using a z-score transformation. Note that before entering the SDS(*t*) and the SPE(*t*) into GLM2, we controlled for the correlation between the two parameters. Results revealed no significant correlation between SDS(*t*) and the SPE(*t*) at the group level (mean *p* = 0.094, mean *r* = −0.368 ± 0.03 SEM).

#### fMRI analysis of the non-social learning task

GLM3 was constructed for the non-social reinforcement-learning task. it was built similarly to GLM1 except that there was no regressor encoding competition. The same procedure was used for the parametric modulators. First, Q(*t* + 1) was normalized using the Fisher transformation and then entered as a modulator of the categorial regressor denoting the outcomes. Similarly, GLM4 was constructed for the non-social reinforcement-learning task. GLM4 was constructed in the same way as GLM2 except that there was no regressor encoding competition in this task. The same procedure was used for the parametric modulators. First, Q(*t*) and PE(*t*) were normalized using the Fisher transformation and then entered as modulators of the categorial regressor denoting the outcomes. The orthogonalization procedure was disabled to give equal “weight” to the parametric modulators and allow them to compete to explain the variance.

All GLM models included a high-pass filter to remove low-frequency artifacts from the data (cut-off = 128 s) as well as a run-specific intercept and 6 motion parameters estimated from the realignment step, to covary out potential movement-related artifacts in the BOLD signal. Temporal autocorrelation was modeled using an AR(1) process. Regressors of interest were convolved with the canonical hemodynamic response function (HRF) using a boxcar that lasted for the duration of the visual stimulus associated with each regressor.

### Computational modeling

To capture behavior, we used 6 variants of the Q-learning model. We compared them using the Bayesian information criterion (BIC) to select the model that best described the data using Bayesian group comparison with the VBA Toolbox on MATLAB [19]. All models were constructed based on a similar algorithm previously described in a previous paper from our group [16]. Only the model presenting the highest exceedance probability using the BIC and the Log-Likelihood (LL) was analyzed. All models are described in the supplementary data section (Table S5 and Fig. S6, computational modeling, estimation and comparison procedure).

To investigate learning in the non-social task in a similar way to that of social hierarchy learning, we decided to use similar models. We also tested two variants of this reinforcement-learning scheme. The results confirmed that the one alpha learning rate is the best model (Table S5 and Fig. S6).

### Correlational analysis

The Pearson correlation requires that the data have a normal distribution. We thus tested for normality of the distribution for each variable entered in the correlation and selected the correct test accordingly to the result. If the normal distribution assumption was met then we performed a Pearson correlation. Otherwise, we performed a Spearman correlation. Comparison of the correlation coefficient were performed using the Fisher’s *Z*-test. It allows testing the significant differences between the correlation coefficient in the social and non-social task between the SERT level in the DRN and the BOLD signal related to the SDS(*t*) and Q(*t*).

## Results

### Behavioral results

Investigation of the choice frequency revealed a main effect of outcome probability (*F*_(2,58)_ = 42.81; *p* < 0.001) and an interaction effect between the outcome probability and task modalities (F_(2,58)_ = 8.89; *p* < 0.001) (Fig. 1A, right panel). *Post-hoc* analyses revealed that participants selected the inferior opponent (*M* = 0.40, SEM = 0.02) more frequently than the intermediate (*M* = 0.30, standard error of the mean, SEM = 0.01; *t*_(29)_ = 3.74, *p* = 0.004) or superior opponent (*M* = 0.29, SEM = 0.02; *t*_(29)_ = 3.33, *p* = 0.04) in the social learning task. Similarly, participants selected the easiest slot machine to win on (*M* = 0.48, SEM = 0.08) more frequently than the intermediate one (*M* = 0.33, SEM = 0.06; *t*_(29)_ = 4.68, *p* < 0.000) and the most difficult one (*M* = 0.18, SEM = 0.03; *t*_(29)_ = 8.82, *p* < 0.001) in the non-social learning task (Fig. 1B, right panel). Also, participants selected the intermediate slot machine more frequently than the most difficult one (*t*_(29)_ = 5.18, *p* < 0.001).

### SERT level in the DRN is linked to the social learning rate

The dorsal raphe nucleus (DRN) has the highest concentration of 5-HT neurons in the brain [17]. It is the main source of serotonin in the cortex and the basal ganglia and is a good candidate for being at the origin of 5-HT regulation for learning the opponent’s social dominance status. First, confirming previous reports [20], a statistical map of the estimated SERT level using the average BP_ND_ showed a large SERT distribution in the DRN and the striatum (Fig. 2A, see “Methods”).

We modeled the behavioral data using a reinforcement Q-learning algorithm. Model RL1, with one learning rate and no updating according to performance was the most likely (called ‘no accuracy monitoring’). For the non-social task, the model with one learning rate was also the most likely (cf. Computational modeling, estimation, and comparison procedures). Participants’ choices and model predicted choices are shown in Fig. 2B. We observed no significant difference in the alpha parameter for the social and non-social tasks (*M* = 0.38, SEM = 0.41, social and *M* = 0.28 SEM = 0.42, non-social). However, the beta parameter in the social task (*M* = 2.70, SEM = 0.87) was significantly lower than that estimated for the non-social task (*M* = 8.01, SEM = 1.39) (*Z*_(29)_ = −3.67, *p* < 0.001 non-parametric Wilcoxon test), possibly reflecting an increased tendency to explore more in the social vs non-social context.

Next, we investigated the relationship between the learning rate (α) and the BP_ND_ of the SERT ligand [^11^C]-DASB, extracted from the DRN using the Automated Anatomical Labelling (AAL3) atlas [21]. A negative correlation was observed between the SERT level in the DRN and the learning rate of social dominance status (*ρ* = −0.366, *p* = 0.046, Spearman correlation) (Fig. 2C). No such correlation was observed between the SERT level in the DRN and the learning rate in the non-social learning task (*ρ* = −0.187, *p* = 0.322, Spearman correlation).

Comparisons of the parameters from the RL model (α learning rate, β inverse temperature) between the social and non-social tasks revealed a significant difference between the inverse temperature. Yet, no relationship was observed between the SERT level in the DRN and the inverse temperature in the social or in the non-social tasks. Because the inverse temperature is linked to exploratory behavior (e.g. challenging a stronger opponent in the social condition despite having learned his strength), we sought to further explore the link between SERT level in the DRN and behavior during the learning of social ranks. To do this, we divided our sample using a median split procedure according to the level of SERT availability in the DRN, resulting in the formation of two groups of 15 individuals: a low (*M* = 1.09 SEM = 0.07) and a high SERT group (*M* = 2.52, SEM = 0.08). Next, we computed a competitive index (cf. methods section) that reflected competitive choices and compared it between these groups as the social hierarchy learning task progressed. When comparing this index including the group (low vs high) and trial bins (1–6, in bins) as factors (Scheirer-Ray-Hare test), we observed a main effect of group (*F*_(1,5)_ = 4.41, *p* = 0.037): low SERT individuals were more competitive than high SERT individuals. No main effect of the bins was observed (*F*_(1,5)_ = 0.620, *p* = 0.698), nor of the interaction effect between bins and group (*F*_(1,5)_ = 0.595, *p* = 0.703). A Mann–Whitney *post-hoc* test showed that the high SERT group made less competitive choices in the last bin (Median rank = 18.87) than the low SERT group (Median rank = 12.13; Mann–Whitney *Z*_U_ = −2.09, *p* = 0.036) (Fig. 2D).

### PET-fMRI results

#### fMRI analysis revealed the positive encoding of dominance status of the opponent during outcomes

**Fig. 3:**
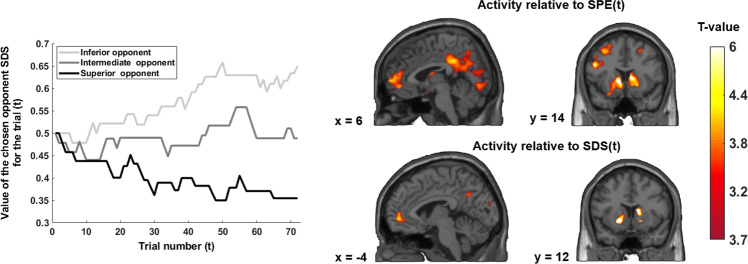
Statistical maps of brain regions tracking the social dominance status of the opponent SDS(*t*) (bottom) and the social prediction error SPE(*t*) (top) at the outcome of the competition. The graph on the left represents the evolution of the SDS(t) for a participant over the experiment. Positive encoding of the social prediction error SPE(*t*) was revealed in the bilateral VS, anterior mPFC, bilateral superior frontal gyrus, and posterior middle cingulate gyrus. Tracking SDS(*t*) engages the bilateral VS and the anterior mPFC. All statistical analyses were performed at a *p* < 0.05 cluster level corrected for Family Wise Error at the whole-brain level, with an initial cluster forming threshold of *p* < 0.001 uncorrected. VS ventral striatum, amPFC anterior medial prefrontal cortex.

We first searched for brain regions encoding the expected value of social victories SDS(*t* + 1), reflecting the social dominance status of the opponent. To do so, we ran a general linear model (GLM1) using SDS(*t* + 1) as parametric regressor. A network of regions including the bilateral VS, the anterior medial prefrontal cortex, and the posterior cingulate cortex coded positively for SDS(*t* + 1) (Table S1). As the social dominance status of the opponent SDS(*t* + 1) can be decomposed into the previous social dominance status SDS(t) and current violation of previous expectations, the social prediction error SPE(*t*), we next sought to disentangle the neural underpinnings of these two components at the outcomes stage. Using GLM2, which includes as parametric modulators SDS(*t*) and SPE(*t*), we performed two one-sample *t*-tests at the group level, one for SDS(*t*) and one for SPE(t). The bilateral striatum, the bilateral superior frontal gyrus, the amPFC, the bilateral angular gyrus and the posterior middle cingulate cortex encoded SPE(*t*) while the bilateral VS and the amPFC encoded SDS(*t*) (Fig. 3, Table S2).

#### DRN level of SERT correlates with the dominance status of the opponent in the ventral striatum

Next, we investigated the influence of 5-HT on learning social dominance hierarchies. We therefore investigated the relationship between the SERT level in the DRN and brain responses related to the dominance value of the opponent (the ventral striatum) during the social dominance hierarchy learning task. We hypothesized that the modulation of the learning rate in social hierarchy learning by DRN levels of SERT should be reflected in the relationship between inter-individual differences in SERT DRN levels and brain regions that encode the dominance value of the opponent. We particularly focused on the VS because it is known to receive large projections from 5-HT neurons [8, 9] and is implicated in the processing of prediction error and expectations [22–24].

**Fig. 4:**
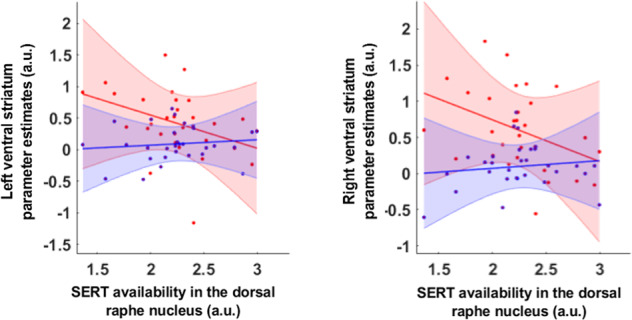
Negative correlation between SERT availability in the DRN and the BOLD response from the ventral striatum tracking opponents’ social dominance status at the outcome of the competition (in red). Significant correlations between SERT availability in the DRN and Ventral Striatum BOLD response related to the tracking of the social dominance status SDS(*t*) during the outcomes of the competitive interaction. No significant correlation was observed between SERT availability in the DRN and BOLD response in the ventral striatum tracking the expected value of the slot machine Q(*t*) (in blue). Moreover, the direct comparison of the correlation coefficient revealed that the correlation coefficients is significantly lower in the social context, compare to the non-social one. VS ventral striatum, SERT serotonin reuptake transporter level, SDS social dominance status, DRN dorsal raphe nucleus.

We first analyzed whether there was a correlation between SERT in DRN and BOLD signal extracted in the bilateral VS related to SDS(*t*), as estimated by GLM2. During the social task, a significant negative correlation between the BOLD signal related to SDS(*t*) and BP_ND_ was observed in the bilateral ventral striatum (*r* = −0.410, *p* = 0.021). We then investigated if the correlation holds in both the left and right VS separately. Results confirmed a significant negative correlation between the BOLD signal related to SDS(t) and BP_ND_ in the left (*r* = −0.383, *p* = 0.037, Pearson correlation test) and right VS (*r* = −0.392, *p* = 0.032, Pearson correlation test) (Fig. 4, Table S3).

Moreover, we also investigated the link between brain activity related to SPE and SERT levels in the DRN. No significant correlation between BP_ND_ in the DRN and the BOLD signal related to SPE(*t*) in the social task was observed (*p* = 0.247, and *p* = 0.153, for the left and right VS respectively, Pearson correlation test).

#### Specificity of the relationship between the SERT level in the DRN and BOLD response related to the social dominance status

**Fig. 5:**
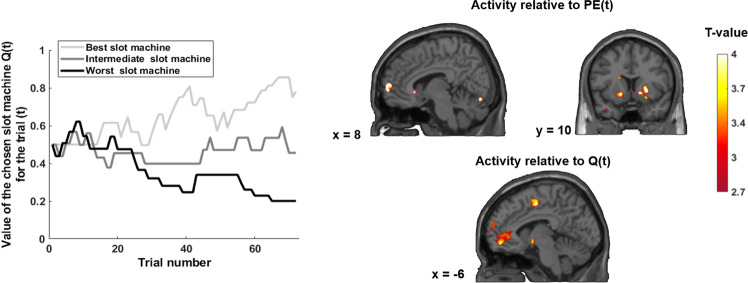
Brain regions tracking the expected value of the slot machine Q(t) and the prediction error PE(*t*) at the outcome. The graph represents the evolution of the Q(*t*) for a participant over the experiment. Tracking Q(*t*) engages the anterior mPFC and tracking the prediction error PE(*t*) engages the right VS, the anterior medial prefrontal cortex, the superior frontal gyrus and the medial posterior cingulate gyrus. All statistical analyses were performed at a *p* < 0.05 cluster level corrected for Family Wise Error at the whole-brain level, with an initial cluster forming threshold of *p* < 0.001 uncorrected. VS ventral striatum, amPFC anterior medial prefrontal.

To investigate the specificity of the correlations observed in the social hierarchy learning task, we performed similar analyses in the non-social learning task. First, using GLM3, we revealed that the amPFC encodes the Q-value of slot machines at *t* + 1, i.e. Q(*t* + 1) (Table S3). Activations are reported at a whole-brain *p* < 0.05, FWE cluster corrected threshold, with an initial forming threshold of *p* < 0.001 (Table S1). Then, using the same approach as for the social task, we decomposed this *Q*-value into Q(*t*) and the current violation of previous expectations PE(*t*). Using GLM4, we observed a similar brain network to the one engaged with social learning encoded Q(t), including the amPFC (Fig. 5 and Table S3) and to a lesser extent the VS (*p* < 0.001 uncorrected for the FWE). A positive PE(*t*) was encoded in the VS, medial PFC, superior frontal gyrus and posterior cingulate gyrus (Fig. 5 and Table S3).

Next, we extracted the BOLD signal related to Q(*t*) and PE(*t*) in the VS to investigate the links between the BOLD signal in these regions and the SERT level in the DRN. Pearson correlation revealed no significant relationship between SERT level in the DRN and the BOLD signal related to the Q(*t*) in the left VS (*p* = 0.547), the right VS (*p* = 0.500) (Fig. 4). Investigating the relationships between the BOLD signal related to PE(*t*) and SERT level in the DRN also revealed no significant correlation (*p* = 0.744, *p* = 0.596, for the left and right VS respectively, Pearson correlation).

Finally, the direct comparisons between the correlation coefficients observed in the social and non-social tasks for SDS(t) and Q(t) respectively, showed significant differences in the left (*p* = 0.049) and the right VS (*p* = 0.046), revealing the specificity of the relationship between BP_ND_ in the DRN and the BOLD response related to the social dominance status in the VS. For illustrative purposes, the BOLD signal in the VS and amPFC are presented in the Fig. S10.

## Discussion

We investigated the links between the neural computations (i.e. tracking and updating signals) required to learn social ranks and serotoninergic activity, reflected by the BP_ND_ of [^11^C]-DASB to SERT in the dorsal raphe nucleus (DRN). Lower levels of SERT in the DRN were linked to higher learning rates during the social task, but no such relationship was observed for the non-social context. When learning social ranks, activity in the ventral striatum and the anterior mPFC tracked both a Social Prediction Error (SPE) related to social victories/defeats and the Social Dominance Status (SDS) of the opponent, representing the total social victories over all successive steps. Moreover, the BOLD signal tracking the opponent’s social dominance status in the ventral striatum correlated negatively with the level of SERT availability in the DRN. This negative relationship only occurred in the social learning context. In addition, individuals with lower SERT BP_ND_ in the DRN showed higher levels of competitive behavior as the task progressed. This suggests a link between low levels of SERT and stronger willingness to engage in social competition.

These results establish a link between SERT availability, the learning of social ranks, and the neurocomputational mechanisms engaged in the integration of long-term social rewards. SERT availability measured using the BP_ND_ of the [^11^C]-DASB is proportional to the SERT density and affinity, which both contribute to serotonin clearance. Thus, low SERT availability likely results in slower clearance of synaptic 5-HT, compared to when there is high SERT availability. With the recent demonstration of enhanced release of serotonin in the synapses after DRN SERT inhibition by selective serotonin reuptake inhibitor [25], we resonated that low SERT in the DRN is associated with a stronger serotonergic signal. However, it may also reflect a lower density of serotonergic synapses where SERT is expressed or lower SERT expression at nerve terminals.

Serotonin has been reported to exert effects on a wide variety of behaviors, including social behavior [26, 27], uncertainty [28], punishment/rewards [29, 30], inhibition of action [29], patience [31–33] and learning [15]. Although diverse, these behaviors are consistent with various aspects of our current findings. Below we discuss how our results add to the current literature on these different 5-HT functions.

### Serotonin, social vs non-social rewards, and unexpected uncertainty

One strength of this study was to compare the SERT-BOLD relationship in social learning relative to non-social learning. The relationship between SERT (measured both in the DRN and the VS, see Fig. S8 for further details) and striatal computation of social dominance status only occurred in the social context. This difference between the social and non-social conditions occurred despite parallel behavioral findings for learning rates and mean RTs (Fig. S2), and also parallel findings for both conditions in engaging a similar vmPFC/ventral striatum network correlating with SPE(t)/PE(t) and SDS(t)/Q(t) (Figs. 3 and 5). A direct comparison of the reaction times between the social and non-social conditions did not reveal a significant difference between the two conditions, nor an interaction effect. It suggests that the differential association of the 5-HT system with social and non-social learning rates is not due to a qualitative difference in decision-making processes as reported in Iigaya et al. for example. The specificity of this relationship to the social context could be due to the fact that when decisions are made in the social context, the degree of uncertainty with respect to the possible outcome increases dramatically because the behavior of other individuals is more difficult to predict than the outcome of a slot machine with fixed payoff probability [34, 35]. A number of theoretical accounts have proposed that unexpected uncertainty (i.e. variability reflecting real changes in the environment) could be encoded by 5-HT [36, 37]. Thus, differences in unexpected uncertainty between the social and non-social task may be explained by the necessity of individuals to track the status of the opponent better, to update that opponent’s status accurately for future trials. In confirmation of this difference between the social and non-social tasks, direct assessment of both the modeled choice entropy and the temperature parameter (beta) showed significant differences between tasks. This reflects higher exploratory choice behavior in the social as compared to the non-social task (Fig. S5). However, no proof of a relationship between the beta parameter and the SERT availability in the DRN was observed. Social decisions may also require more long-term computations of social expected value, in line with recent optogenetic results revealing that 5-HT helps learning for long-term associations only, but not for short-term associations [15]. This differential relationship between the learning rate and the serotoninergic system might be due to a difference in uncertainty about social versus non-social hidden state [38]. Another factor potentially explaining this observed difference is the perceived control that participants may express in both contexts. Serotonin may be more involved in the social than in the non-social context in meeting the individual’s need for control, and its involvement may be dependent on this. Further investigations are needed. A final explanation is that the social hierarchy task may be more motivating than the non-social task to win the social interaction. There could also be an enhanced feeling of punishment when experiencing a social defeat as compared to simply experiencing a monetary loss.

### SERT level and ventral striatum encoding the dominance status

The computational signal of the social dominance status of the opponent in the ventral striatum can be interpreted as the cumulative prediction error that reflects the history of the participant’s choices (Fig. 4). This status of the selected option is updated, based on the previous dominance status of the opponent SDS(t) and the current social prediction error SPE(t). Such encoding of the expected opponent’s dominance status is biologically relevant since this signal conveys information about the value of the previous choice of opponent to guide future choices. Electrophysiological recordings have indicated that neurons in the ventral striatum encode such expected values in rats and non-human primates [39, 40]. Consistent with computational theories of serotonin, our findings indicate that ventral striatal computations of the social dominance status of the opponent are related to SERT availability in the DRN (Fig. 4) [41, 42]. Tonic serotonergic signals have been proposed to reflect the long-term average reward rate as an average RL algorithm [29, 41]. However, serotonin levels may also indicate how beneficial the current environment feels to the animal [14, 42, 43]. Generally, in reward RL algorithms, actions chosen to optimize the expected value optimize the long-term average reward received per time step, and not the cumulative reward received over a finite time window. Recently, the concept of “beneficialness” has been developed, based on optogenetics and electrophysiological recordings from the DRN of freely behaving animals. It supports the theory that the firing rate of DRN 5-HT neurons increases until the outcome is experienced, and is relative to the overall amount of reward earned during the previous trials [14]. The cumulated prediction error, reflects how much a particular option has been rewarded, and could relate to accumulated evidence of how beneficial an option is. Thus, the link between SERT availability and striatal activity related to the dominance status of an opponent establishes, for the first time in humans, a relationship between a computational role of 5-HT and local computations of dominance status of the opponent signal in the ventral striatum.

### Limitations

Our results revealed the mechanism that underlies the establishment of social dominance hierarchies through competitive interactions. Although we considered that each opponent’s profile was of a fixed strength over the course of the experiment (one stronger, one equal and one weaker), in real life situations social hierarchy can be unstable and interact with the current status (e.g. low or high rank) as well as with on stress [44–46]. In this study, we examined the formation of the social dominance hierarchy while participants were learning the strength of the opponent. This is a common situation that occurs when an individual enters a group for the first time. However, we acknowledge that winning and losing, and the neurobiological processes involved in such a context, might be different from winning and losing in an established social dominance hierarchy. Further comparative studies are needed. Also, changes in volatility of the dominance profile might change the learning rate dynamics [47]. Moreover, while being a major force that shapes the hierarchy of our social group, dyadic social competitive interactions are not the only way to access the highest rank within a group of individuals. Social hierarchy can also be learned by observation of others’ interactions without participating in these interactions directly [3, 48] and complex collective dynamics can participate in their stabilization or destabilization through the formation of coalitions [49, 50]. While simultaneous PET-fMRI acquisition is a powerful tool to investigate the relationship between the serotonergic system and brain activity, our sample size is relatively small (*n* = 30) which may limit its sensitivity to weaker statistical associations between 5-HT and behavior. Also, because our results are correlational, neuropharmacological studies will be needed to determine causal links between serotonin manipulation and BOLD signal in the context of learning hierarchies. Also, due to the correlational nature of the results, investigating the causality using pharmacological approaches is needed to confirm the observed effects. Finally, the results are valid only for the healthy male population. Generalization of these results in a female sample is something that needs to be done. We decided to use male only for two reasons. First to investigate the complex relationship between serotonergic system and the establishment of the social dominance and limit confounding factors such as the use of a treatment in patients that interacts with the serotonergic system or hormonal changes in young women related to the menstrual cycle. Second, it offers an opportunity to replicate previous findings and to maximize the overlap between the two studies to draw more comprehensive and cross-sectional conclusions.

To conclude, our combination of computational modeling with simultaneous multimodal neuroimaging PET-fMRI approach suggests a relationship between the role of serotonin signaling and the neurocomputational basis of social dominance hierarchy learning during competitive interactions and highlight the differences with a non-social learning.

## Supplementary information

Supplementary information
