# Supplementary material for: Regulation of social hierarchy learning by serotonin transporter availability
Source: Neuropsychopharmacology. 2022 Aug 9;47(13):2205–12. doi: 10.1038/s41386-022-01378-2 (PMC9630526; doi:10.1038/s41386-022-01378-2)
Supplement: Supplementary file 1 — Supplementary information [file 41386_2022_1378_MOESM1_ESM.pdf]

Supplementary information

Supplementary material

Cover story

In the social dominance hierarchy learning task, participants were led to believe that they were competing against three other participants anonymously connected online (**Figure 1.A**). To ensure that they believed in this interaction with others, they were trained outside of the scanner on a perceptual decision-making task. The training started with a fake connection to the behavioral room where the opponents wait. The first connection failed and the experimenter call the other experimenter in charge of the opponents to solve the issue. The failure was manipulated by the experimenter to increase the belief of the participants that another experimenter is also engaged in the experiment to give instructions to the opponents. Then, the second attempt to connect to the behavioral room succeeded and the participants were able to see other people waiting in the room while the other experimenter gave some instructions. After this brief video (recorded for purpose and stopped by the experimenter to start the training), the participant began the training. In this task a participant has to indicate within 1 s whether the majority of arrows point to the left or right direction on the screen, using a left/right button press. This training period included (non-social) feedback based on trial performance (red fixation cross = incorrect decision, green cross = correct decision, and yellow cross = no answer given). They were told that the opponent trained themselves in the behavioral room as they were doing.

Following this training, participants were told that they had to compete in real-time with the other individuals using the perceptual decision-making task and received the following instructions: ‘‘If both responses are correct, the fastest player wins. If one player gives an incorrect response, the accurate player wins. If both responses are incorrect, the slowest player wins. If one player doesn’t respond, then he loses automatically.’’ Then the participant was placed on the scanner. After the anatomical scan, a new connection to the behavioral room was performed and the first social dominance hierarchy learning task began.

Scanning procedure

The stimuli were presented with a screen resolution of 1280×1024 pixels, displayed at a visual angle of 24-18°, centered on the screen. The participants were asked to use their index and middle fingers of the same hand to respond by pressing a 4-button controller. Stimuli were presented, and the responses to the stimuli were collected using the Psychotoolbox Toolbox Version 3 (PTB-3) on MATLAB (version 7.16.0, R2013a, Natick, Massachusetts: The MathWorks Inc). Neuroimaging was performed at the CERMEP Center (Lyon, France). Before the bolus injection, the anatomical image was acquired. Then after the bolus injection, the participant rested for 10 minutes before starting the task. The PET-fMRI experiment consisted of two social dominance hierarchy learning tasks of approximately 15 min each followed by a rest of 5 min, separated by a non-social learning task and followed by a post-task rating to assess learning.

PET and fMRI preprocessing

PET data acquisition

PET data were acquired in list-mode, over 90 min. The acquisition started with the intravenous injection of a bolus of [^{11}C]-DASB, a radiotracer that binds SERT. Mean [^{11}C]-DASB injected activity (Mean=268.3MBq, SEM=7.3MBq). PET data were submitted to list mode motion correction 53, then re-binned into 24-time frames (variable length frames, 8 x 15s, 3x60s, 5x120s, 1x300s, 7x600s) for dynamic reconstruction [1,2]. Images were reconstructed using OP-OSEM 3D incorporating the system point spread function using 3 iterations of 21 subsets. Sinograms were corrected for scatter, random, normalization, and attenuation 53. Reconstructions were performed with a zoom of 2 yielding a voxel size of 1.04×1.04×2.08 mm^3 in a matrix of 344 × 344 × 127 voxels. Gaussian post-reconstruction filtering (FWHM=2mm) was applied to PET images.

PET preprocessing and kinetic modeling

Average PET image was computed for coregistration purposes. Anatomical T1 MPRAGE was coregistered (rigid transform) onto the average PET image. Regional labeling of the brain structure was performed with the Hammersmith 83 regions atlas [3]. This allowed us to extract regional time activity curves based on the subject space, by coregistering the atlas on the subject space and performing the extraction. Parametric images of BP_{ND} were computed by applying the Simplified Tissue Reference Model (SRTM) [4] and using cerebellar grey matter as a reference region assumed to be devoid of SERT transporters [5]. PET images were then spatially normalized into the standard Montreal Neurological Institute (MNI) atlas space using DARTEL (diffeomorphic anatomical registration through exponentiated lie algebra) toolbox procedure, using the T1 SPM template and resulting in voxels of 2 x 2 x 2 mm [6]. These statistical maps, representing the SERT level, were used to extract BP_{ND} from our region of interest.

We performed a bolus injection of [^{11}C]-DASB at the beginning of the acquisition. [^{11}C]-DASB is a tracer frequently used to assess the SERT levels. The entire acquisition lasted 90 mn [7]. We first allowed the [^{11}C]-DASB to bind SERT while participants were lying in the PET-fMRI scanner. Subjects performed the social hierarchy learning and non-social learning tasks after the anatomical scan and a resting-state period of 10 minutes. The binding potential of [^{11}C]-DASB is thought to reflect serotonin activity at the brain level [8]. However, one must be cautious when interpreting the results produced by [^{11}C]-DASB. Indeed, this tracer only marks the level of the free serotonin reuptake transporter. Two studies conducted in humans have shown no modification of the BP_{ND} after tryptophan -a serotonin precursor- depletion inducing a reduction of 85% in plasma levels of tryptophan [9,10]. As confirmed by several studies, tryptophan depletion does robustly lower the brain tryptophan level, resulting in reduced brain serotonin level [11,12]. Since no modulation of BP_{ND} was observed after such tryptophan depletion, it is very unlikely that a physiological change in serotonin, such as serotonin release, would result in a change in BP_{ND} of the [^{11}C]-DASB [13].

MRI data acquisition

All functional MRI acquisitions were performed using EPI BOLD sequences. Functional scans were performed using the following parameters, single-shot EPI, TR / TE=2400/34, flip angle 85 °, 52 axial slices interlaced, 2 mm thickness, 2 mm gap, FOV=192x192x125. Volumes were collected, in an interleaved manner. The first acquisition was performed after stabilization of the signal. Anatomical MRI acquisition consisted of 3D sagittal T1-weighted sequences, repetition

time=2300 ms; echo time=2.34 ms; flip angle=8; field of view=256 mm; voxel size=1 x 1 x 1 mm3. The anatomical volume covered the entire brain using 256 adjacent slices of 1-mm thickness.

fMRI data preprocessing

Image analysis was performed using SPM12 (Wellcome Department of Imaging Neuroscience, Institute of Neurology, London, UK, fil.ion.ucl.ac.uk/spm/software/spm12/). Time-series images were registered in a 3D space to minimize any effect that could result from participant head-motion. Once DICOMs were imported, functional scans were corrected for slice timing, realigned to the first volume and corrected for motion displacement. Structural images were previously co-registered on the average Dynamic PET image that had been computed. This procedure ensured that functional images were in the same space as PET images. Finally, to perform group and individual comparisons, EPI Images were co-registered with structural maps and spatially normalized into the standard Montreal Neurological Institute (MNI) atlas space using DARTEL toolbox procedure, using the T1 SPM template [6]. Images were then spatially smoothed with an 8 mm isotropic full-width at half-maximum (FWHM) Gaussian kernel using the standard procedures in SPM12. Note that all the following general linear models (GLM) described also included motion parameters as regressors of no interest, and two session constants (representing our four runs and accounting for its effect) were added for GLM of the social task.

ROIs definition

We studied the relationship between SERT levels and the BOLD signal related to both the expected value Q(t) or SDS(t) and the social prediction error SPE(t). Previous literature revealed the involvement of the ventral striatum in the computation of expected value and prediction error. We defined two ROIs using an anatomical definition of the nucleus accumbens based on MNI space from the Automated Anatomical Labeling atlas [14]. The search volume was defined by an ROI of the left VS and another for the right VS. We used the DRN definition from the same atlas to extract the SERT level for the DRN in the statistical map of the BP_{ND} representing the SERT level. In addition to the striatum, the anterior medial prefrontal cortex is also known to be engaged in the tracking and processing of the value. Before any extraction, ROIs were resampled in SPM12 to match the size of the voxels and images of the MRI and PET acquisitions (already co-registered together). Extraction of the BOLD signal was conducted at the single level subject estimated signal within the ROIs defined and using MarsBaR toolbox for MATLAB [15].

Computational modeling, estimation and comparison procedures

Reinforcement Learning model of the social dominance hierarchy learning task

The models assumed that the probability of choosing to compete with opponent i over another opponent j depends on the relative difference in the social dominance status (SDS) of that opponent *versus* the other opponent (both being presented on the screen) (Equation 1). This relationship defines the softmax decision rule:

$$p(i) = \frac{\exp(\beta * SDSi)}{\exp(\beta * SDSi) + \exp(\beta * SDSj)} \qquad \text{(Eq. 1)}$$

This equation defines the stochastic decision rule (softmax) that calculates the probability p(i) of choosing the opponent i given the other opponent j. β is the inverse temperature parameter. It is a free parameter that dictates to what extent the decision is deterministic relative to the SDS dominance status of the available opponent (β was constrained in the interval [0 +∞]).

Then, according to the choice of the participant, the social dominance status of the opponent selected was updated following the exponential, recency-weighted average algorithm. The free parameter β is the inverse temperature parameter and dictates to what extent the decision is deterministic relative to the SDS social dominance status of the available opponents i and j. β was constrained to be positive, and a β of 0 represented the most exploratory behavior (random choice), whereas a high β denotes a highly exploitative behavior by which the participant prefers to compete with the weaker opponent.

$$SDSi(t+1) = SDSi(t) + \alpha(\mathrm{R(t)} - SDSi(t)) \qquad \text{(Eq. 2)}$$

$$SDSi(t+1) = SDSi(t) + \alpha\mathrm{SPE(t)} \quad \text{(Eq. 3)}$$

Equation 2 assumes that the social dominance status of the selected opponent is updated according to his previous social dominance status and the differences between the actual reward R(t) and his ongoing social dominance status SDS(t). This difference is modulated by the free parameter α, which represents the learning rate of the model and was constrained between 0 and 1. The social prediction error SPE(t) is the difference between the reward at time t from the current social dominance status of the opponent SDSi(t) resulting from the ongoing competitive interaction (reward was arbitrarily set to 0 for a defeat and 1 for a victory). SDSi reflects the cumulative social reward prediction error and is called the social dominance status of the opponent. This definition allows us to model that participants mostly preferred to compete against the inferior opponent, after probing the other opponents' strengths, to avoid social defeats. In fact, defeating an opponent will increase the relative social dominance value of that opponent and losing will decrease it. α represents the subject's learning rate (between 0 and 1 and is assumed to be the same for defeats and victories). This allowed us to investigate the neural correlates of both the SPE(t) and the SDS(t) dominance status of the selected opponent in the current trial. Note that SDSi was updated even if the participant did not choose that opponent, because the participant might choose them as an adversary in a future trial, and update their value. There was an exception to this with three participants who systematically chose not to play against one of their three opponents during the entire first block of the social dominance learning task. These blocks for these participants were therefore entirely removed from the analysis.

Because performance of the perceptual decision-making task could affect the updating of the social dominance status of the opponent (SDS(t)), we constructed three different families of models. The first family included RL3 and RL6 and followed the updating rule as defined by Equation 2. The second family, including RL1 and RL4, did not update the social dominance status of the opponent (SDS(t)) after incorrect answers to the perceptual decision-making task, (ACC monitoring). The last family, including RL2 and RL5 models, did not update after incorrect answers and included a performance-weighting parameter ω (Equation 4 and 5 for a victory and a defeat respectively) (ACC monitoring, weighted RT).

$$SDSi(t+1) = SDSi(t) + \omega * (1 - \mathrm{Perf}) * \left(\mathrm{R(t)} - SDSi(t)\right) + (1 - \omega) * \alpha * (\mathrm{R(t)} - SDSi(t))$$

(Eq. 4)

$$SDSi(t+1) = SDSi(t) + \omega * \text{Perf} * \big(\text{R(t)} - SDSi(t)\big) + (1-\omega) * \alpha * (\text{R(t)} - SDSi(t))$$
(Eq. 5)

The ω is the performance weighting parameter, with a higher value of ω reflecting a higher effect of the performance, Perf, on the prediction error. It represents how sensitive a participant is to his own performance, and updates the dominance value of the opponent according to the participant's performance. Perf is the normalized performance of the participant on the current trial and is computed as:

$$Perf = 1 - \frac{\log(RT_t) - \min(\log(RT_t))}{\max(\log(RT_t)) - \min(\log(RT_t))} \qquad \text{(Eq supp. 6)}$$

Note that the variant of the models used varies according to the update of the social dominance status of the opponent (SDS(t+1)). Furthermore, we created two distinct families according to the learning rate for each model defined above (no updating, accuracy monitoring, and accuracy monitoring weighted by the reaction time). A first group of models used the same alpha learning rates for victories and defeats and included RL1, RL2 and RL3. The remaining models, RL4, RL5 and RL6, updated the social dominance status of the opponent (SDS(t+1)) according to two different alpha learning rates, one for victories the other for defeats. For the models with two alpha rates, the probability of choosing one opponent *i* to bet on, over another opponent *j* was defined with the same softmax decision rule (Eq supp 1). Here again, the difference concerns the value updating. Compared to the first model RL1 there were two different learning rates, one for victories and one for defeats:

$$SDSi(t+1) = SDSi(t) + I * \alpha_{win} * \text{SPE(t)} + (1 - \text{I}) * \alpha_{loss} * \text{SPE(t)} \qquad \text{(Eq. 7)}$$

Where I=1 if the participant won and 0 if he lost. It assumed that participants learned differently after experiencing a victory or a defeat using a dual asymmetrical learning rate. Estimation of optimal parameters and goodness of fit were performed on the subject level using the Variational Bayes Approach proposed by [16] and implemented in a validated MATLAB toolbox.

Reinforcement Learning model of the non-social learning task

To investigate the neural processes underlying learning, we used a Q learning model RL1, and estimated the learning rate and inverse temperature of each participant. This model assumed that the probability of choosing one slot machine i to bet, over another slot machine j is highly related to the difference in their internal expected values (Qi and Qj).

$$\text{p(i)} = \frac{\exp(\beta * Qi)}{\exp(\beta * Qi) + \exp(\beta * Qj)} \qquad \text{(Eq. 8)}$$

This equation defines the stochastic decision rule (softmax) that computes the probability p(i) of choosing the slot machine *i* given the other *j*. β is the inverse temperature parameter. It is a free parameter that dictates to what extent the decision is deterministic, relative to the values of the available options (β ∈R).

Using this RL algorithm, we modeled the dynamics of the expected value of the slot machine i (Qi) and how it varies according to the feedback received during the outcome stage following the gamble at time *t*:

$$Qi(t+1) = Qi(t) + \alpha PE \quad \text{(Eq. 9)}$$

R is the “reward” resulting from the ongoing slot machine and was arbitrarily set to 0 for a monetary loss and 1 for winning money. α represents the learning rate of the model. It is constrained between 0 and 1 and is equal for both loss and win. Qi(t) represents the expected value of the chosen slot machine at the outcome and reflects the weighted cumulative prediction error. This procedure enables us to investigate the neural correlates of the chosen value at the outcome. It is important to note that during modeling, Q was updated if the participant did not choose a slot machine.

Behavioral analysis

We excluded trials where a participant did not choose at the time of selection of the opponent or of the slot machine. These trials represent less than 3% of all trials and the results remain similar even when they are included in the analyses. Such trials were considered as missed choices.

All statistical analyses were performed using SPSS v21.0 (SPSS Inc., Chicago, IL, USA). Normal distribution was assessed with a Shapiro-Wilk test and visually. If data distribution was not normal, we performed a Friedman test, otherwise a repeated measure ANOVA was conducted. Then, we ensured that homoscedasticity of variances was respected using a Mauchly test. If not, we applied a Greenhouse-Geisser correction to our ANOVA. For multiple comparisons, a *post-hoc* comparison (with Bonferroni correction) was conducted according to the previous test used.

Concerning the correlation analyses, if data were normal, we performed a Pearson correlation, otherwise, Spearman correlation was conducted. In addition, correlation coefficients from the social dominance hierarchy learning task and the non-social learning task were compared using a single side test of correlation comparison [17].

Behavioral scale

At the end of the experiment, participants completed a series of questionnaires aimed at assessing different aspects of personality. To assess anxious temperament, participants completed the Spielberger trait and state anxiety scale (Y-T and Y-S version) [18]. A distinction is made between the trait (YT), which is a general temperament, and the state (YS), which is more variable over time and corresponds to the person's current temperament. To measure depressive temperament, participants completed the BECK scale [19]. Participants also completed the BIS-BAS questionnaire, which assesses two general motivational systems underlying behavior: the behavioral inhibition scale (BIS) and the behavioral approach system (BAS) [20]. Social assertiveness was assessed using the assertiveness questionnaire [21]. Finally, a scale to assess the social orientation of individuals was also completed. Social orientation was assessed using the social dominance orientation scale [22]. All demographic data are summarized in the supplementary data (**table S6**).

Confidence rating prediction

For the mixed effect linear regression explaining the confidence rating, we included several explanatory variables: the trial number, the reward probability (represented by the opponent or slot machine selected), the reaction time of the choice, the BP_{ND} in the DRN, the block number and the task condition (social or non-social). The trial was coded as the trial number when the confidence rating was requested during the task (approximated with a continuous variable). The reward probability categories were coded for both social and non-social tasks as 3=superior or worst, 2=intermediate or middle and 1=inferior opponent or best (nominal variable). The logarithm of the reaction time was for each decision (opponent or slot machine) selection (0.23 ± 0.37s, continuous variable). The BP_{ND} was the average level of SERT within the DRN for each participant (continuous variable). The block number was coded as 1 for the first block of the social learning task and the block of non-social learning and 2 for the second block of the social learning task (nominal variable). Finally, the task condition was coded as 1 for the social task and 2 for the non-social task (nominal variable).

Comparison of social and non-social brain activities related to the expected value and prediction error

A last GLM, GLM5 was constructed to compare the social and non-social brain activations related to the computation of the expected value and the prediction error. This GLM includes the same regressors modulated by the same parameters as GLM2 and GLM4 (for both social and non-social tasks respectively). We constructed a contrast at the subject level accounting for the fact that the social task includes two runs whereas the non-social task only includes one run as follow [1/2 -1 1/2]. We then investigated the differences at the group level using a one-sample t-test on the previously define contrast.

Supplementary results
Reaction times

Concerning the reaction time for the opponent selection, we first z-scored all reaction times and then performed a one-way repeated measures ANOVA by pooling results from the two blocks of competition. The results revealed significant differences in the speed of opponent selection ($F_{(2,52)=}$13.35; p<0.001) (Figures S1B). The *post-hoc* tests revealed that participants were faster when selecting inferior opponents (M=-0.12, SEM=0.03) compared to the intermediate (M=0.13, SEM=0.04; $t_{(31)}$=-4.35, p<0.001) and the superior opponent (M=0.17, SEM=0.06; $t_{(31)}$= -3.95, p<0.001). No significant differences were observed between the reaction times to select the intermediate and the superior opponent (paired $t_{(31)}$= -1.29, p=0.205).

Concerning reaction times for the perceptual competition, we first z-scored all the reaction times and then performed a one-way Repeated Measure ANOVA by pooling results from the two blocks of competition. The results revealed no significant difference in the speed of decision for the perceptual decision-making stage during the competition ($F_{(2,52)=}$0.12; p=0.94) (**Figures S1C**).

We also compared the reaction time of the opponent selection (concatenate across the two blocks of social learning task) with the reaction time of the slot machine selection. To do so, we performed a repeated measure ANOVA with the task condition (social vs non-social) and the opponent/slot machine reward probabilities as factors. The result revealed a significant main effect of the opponent/slot machine ($F(_{2,58})$=26.167; p=0.000). Participants were faster at selecting the Inferior opponent (or best slot machine, M=-0.159, SEM=0.026) compared to the Intermediate (p<0.001, M=0.164, SEM=0.033) and compared to the Superior (p<0.001, M=0.283, SEM=0.057). There was no main effect of the condition (social vs non-social task) ($F(_{2,58})$=3.457; p=0.063), and no interaction effect between the condition and the reward probabilities ($F(_{2,58})$=3.159; p=0.051).

Selection of opponents in the social dominance hierarchy learning task: control for block effects

During the two independent competition tasks, participants were told to choose with which opponents they wanted to compete within each trial. The two-way repeated-measures ANOVA with a Greenhouse-Geisser correction, including opponent and competition blocks as factors of interest, revealed significant differences in the choice frequency of the participants according to the opponent $F_{(2,52)=}$7.16; p=0.005) (**Figure S1A**). The *post-hoc* analysis revealed that participants selected the inferior opponent more (M=0.40, SEM=0.02) compared to the intermediate (M=0.29, SEM=0.01, $t_{(1,29)=}$3.33; p=0.001) and the superior opponent (M=0.27, SEM=0.01; $t_{(1,29)}$=3.75; p=0.004). No significant differences were revealed between the choice frequency for the intermediate and the superior opponent ($t_{(1,29)=}$1.12; p=0.22). There was no significant interaction effect between block and opponent types ($F_{(2,52)}$=0.28; p=0.75) (Figures S1A).

We also performed a similar analysis on reaction time (RT) for the opponent selection, adding the block as a factor of interest into a two-way repeated measure ANOVA. The Greenhouse-Geisser corrected results from this ANOVA, revealed a significant effect of the opponent ($F_{(2,58)}$=14.76; p<0.001). *Post hoc* tests shown that during the first block of competition, participants were faster in selecting the inferior opponent (M=-0.17, SEM=0.04) compared to the intermediate (M=0.11, SEM=0.06; $t_{(29)}$= -3.56, p<0.001) and the superior opponent (M=0.25, SEM=0.06; t(31)= -4.57, p<0.001). They were also faster in selecting the intermediate opponent compared to the superior (t(29)=-2.29, p=0.029). During the second block of competition,

participants were faster in selecting the inferior opponent (M=-0.08, SEM=0.04) compared to the intermediate (M=0.16, SEM=0.2; paired t(29)=-3.95, $p<0.001$). No significant differences were observed between the reaction time for selecting the intermediate opponent compared to the superior opponent during the second block. No effect of the block was revealed ($F_{(2,62)}=0.073$; p=0.930), yet we observed a significant interaction effect between the opponent and the block ($F_{(2,58)}=5.32$; p=0.01) (**Figure S1B**).

Also, statistical analysis of the reaction times at the perceptual decision making task (PDM) revealed no significant opponent effect ($F_{(2,58)}=0.08$; p=0.92), and no block effect across the three different opponents ($F_{(2,58)}=0.49$; p=0.48) or an interaction effect ($F_{(2,58)}=0.08$; p=0.17) (**Figure S1C**).

Dorsal Raphe Nucleus predicts the confidence rating during social and non-social learning

Mixed-effects linear regression was computed to predict the confidence of victories for each of the three opponents (social) or slot machines (non-social) during the tasks. To do this, we selected potential explanatory variables and entered them as predictors of the confidence rating. The explanatory variables were: the trial number of the confidence rating (β1= Trial), the reward probability categories for the opponent or slot machine types (β2= Reward Probability), the outcome of the previous trial (β3= Previous Outcome), the log(RT_{rating}) of the confidence rating (β4= RT_{rating}), the log($RT_{opponent}$) of the opponent selection (β5= $RT_{opponent}$), the BP_{ND} in the DRN (β5= BP DRN), the block of social dominance hierarchy learning through competition (to control for an effect of the block; β7= Block), and the conditions, social vs non-social (β8= Condition). A significant effect was found ($F_{(3,1000)}=22,88$; p<0,001; Durbin-Watson=1,347), with an R^2 of 0.165. The trial number of the confidence rating, the reward probability categories, the previous victory/reward, the log(RT_{rating}) for the confidence rating, and the BP_{ND} of the DRN were all significant predictors of the level of confidence (**Figure S4B, table S5,** regression model comparison). Participant's predicted confidence rating is equal to:

$$[\text{Confidence rating}] = \varepsilon + \beta1*\mathbf{Trial} + \beta2*\mathbf{Reward\ Probability} + \beta3*\mathbf{Previous\ Outcome} + \beta4*\mathbf{RT_{rating}} + \beta5\mathbf{RT_{opponent}} + \beta6*\mathbf{BP_{ND}} + \beta7*\mathbf{Block} + \beta8*\mathbf{Condition} \quad \text{Eq. 10}$$

Where ε represents the error term (ε=61.47±6.39), and βi are the estimated parameters of the variables explaining the confidence rating. The results show that higher BP_{ND} in the DRN leads to greater confidence (β6=3.19±1.54, p=0.039). Subjects also tend to be more confident throughout the task (β1=0.19±0.03, $p<0.001$) but as expected this confidence decreased as the probability of winning against the opponent or the slot machine decreased (β2=-7.21±0.79, $p<0.001$). Surprisingly, subjects were more confident when they took a longer time to rate their confidence level (β4=2.69±0.97, p=0.006) and they were also less confident following a previous victory (β3=-3.86±1.21, p=0.002). The block number, the task condition, and the log($RT_{opponent}$) for the opponent selection did not explain the confidence rating (p=0.662, p=0.577, and p=0.339 for the block, task condition, and RT for the opponent selection respectively). We also plotted confidence ratings and winning probabilities to illustrate time variation in confidence ratings for the social and non-social tasks (**Figure S4C**).

Score of the reward responsiveness scale correlates to the SERT availability in the DRN

The SERT availability in the DRN and individuals' score on the BIS/BAS personality questionnaire revealed a positive correlation with the reward responsiveness subscale (p=0.019, r=0.427). Higher SERT availability in the DRN was associated with higher reward responsiveness. No correlation between SERT availability and other subscales of the BIS/BAS were observed, and no other relationships were observed with the other personality scales (**Figure S7**).

Additional investigation of the link between the SERT level in the DRN and the parameters of the models

Investigating the correlation between the inverse temperature parameters and the SERT level in the DRN revealed no significant correlation in both the social task (r=0.145, p=0.445, Spearman correlation) and the non-social task (r=0.155, p=0.413, Spearman correlation). Additionally, a negative correlation was observed between the learning rate and the inverse temperature for both the social (r=-0.393, p=0.032, Spearman correlation) and non-social tasks (r=-0.752, p<0.001, Spearman correlation).

As individuals expressed more or less competitive behavior at the end of the social dominance hierarchy learning task, we thought that the correlation between the learning rate and the BP_{ND} in the DRN could be affected by this behavior. We thus performed fitting excluding the last bin of the task (corresponding to the last 12 trials) and correlated the alpha learning rate obtained with the BP_{ND} in the DRN. One-tailed Pearson correlation revealed a significative negative correlation, with a higher SERT level (assessed with the BP_{ND}) associated with a lower learning rate (r=-335, p=0.035).

In addition, we have shown that Low BP_{ND} individuals tend to persist in the competitive behavior through the task compare to high BP_{ND} individuals. We thus tested whether model frequencies were the same between groups (Low vs High BP_{ND}) created according to the SERT level in the DRN. To this end, we used the random-effect analysis implemented in the Variational Bayes Approach (VBA) toolbox. The results revealed no difference in the model frequency, showing that the same model explained behavior for both the low and high SERT groups.

Local ventral striatum level of SERT correlate with the dominance status of the opponent

We also investigated the relationship between the VS BOLD response in the social learning task and local SERT availability not from the DRN but the VS using a predefined ROI from the AAL3 atlas (**Figure S8**). A negative correlation was observed between the VS BOLD signal related to SDS(t) and the local SERT availability (r=-0.368, p=0.045 and r=-0.401 and p=0.028 for the left and right VS respectively). No correlation was observed between the local BP_{ND} and the BOLD signal related to SPE(t) in the social task (p=0.118 and p=0.098 for the left and right VS, respectively).

Analysis performed in the non-social task revealed no correlations between BOLD related to Q(t) signal and SERT availability in the left (p=0.085) or right VS (p=0.610). Comparison of the correlation coefficients between the BP_{ND} and the BOLD signal in the VS obtained in the social

and non-social task showed lower correlation coefficients for the social task (right VS: r=-0.401; left VS: r=-0.368) than that for the non-social task (right VS: r=0.246, p=0.004; left VS: r=0.092, p=0.047) (**Figure S8**).

In addition, we extracted the BP_{ND} in the left amygdala and correlate it to the activity related to the BOLD signal tracking the SDS in the VS. Spearman correlation revealed no significant correlation between the BOLD signal in the left and right VS and the BP_{ND} of the amygdala (p=0.103 and p=0.437 for the right and left VS respectively). It highlights the specificity of the relationship observed between the brain activity involved in the SDS and the BP_{ND} of the DRN and ventral striatum.

Comparison of social and non-social brain activities related to the expected value and prediction error

The comparison revealed a significantly greater signal in the SDS(t) compared to Q(t) in the right middle temporal gyrus, the left and right middle frontal gyrus, the right superior parietal lobule, the right caudate and the right inferior frontal gyrus.

Comparison of the brain activity related to the social prediction error SPE(t) and the prediction error PE(t) revealed significant differences in the putamen. Stronger activity is observed for the social prediction error compare to the prediction error (**Figure S9**). All statistical analyses were performed at a $p<0.05$ cluster level corrected for Family Wise Error at the whole-brain level, with an initial cluster forming threshold of $p<0.001$ uncorrected.

Supplementary discussion

Exploration or exploitation behavior?

When participants were separated into two groups according to low or high SERT levels in the DRN (low and high BP_{ND}), we observed decreasing levels of competition as the social task progressed in the high BP_{ND} group (**Figure 2D**). These participants would be expected to be less sensitive to extracellular 5-HT as an increased SERT availability is hypothesized to be associated with an increased 5HT reuptake and fewer 5-HT release in the nerve terminal. This effect may be related to a role of serotonin in favoring persistence of current behavior: that is, low BP_{ND} in 5-HT neurons of the DRN may favor the persistence of a default choice to compete. Likely, a recent optogenetic study proposed that the reason that 5-HT stimulation favors patient waiting is because it favors persistence of a current behavior, even if it is active [23]. In our experiment, the default behavior was to try to win the competition in the social task, even after relative social dominance status has been learned. At the end of the task, lower BP_{ND} in the DRN, which presumably resulted in higher levels of extracellular 5-HT, or greater sensitivity to extracellular 5-HT, favored persistence in selecting the strongest opponent, even when the alternative option (to play against a weaker opponent) is more likely to lead to a social victory.

Time-scale of the relationship between ventral striatum activity and BP_{ND} DRN

The ventral striatum encoded both social dominance status (SDS) and social prediction error SPE at the outcome. Yet, only ventral striatum related social dominance status computation, but not SPE, correlated with SERT availability in the DRN, consistent with the time-scales of both the PET measurement (i.e. one BP value per subject for a given brain region) and with the social dominance status computation reflecting the incorporation of future social outcomes over long periods. The 5-HT system is known to participate in a variety of cognitive processes at different time scales, including slow time-scale cognitive processes such as motivation, mood, and learning [24–26]. Recent findings also reveal sub-second serotonin fluctuations which may be opponent to dopamine [27], showing positive transients to negative reward PE and negative transients to positive reward PE [28,29]. In humans, methods such as PET or pharmacological approaches are on the timescale of minutes and cannot resolve the sub-second computations supported by fast neuromodulation [30]. These approaches are complementary as neuromodulators such as 5-HT can signal over more than one timescale, with partially separable tonic and phasic activity, and different receptor types sensitive to different timescales.

Relationship between 5-HT and choice confidence

Bayesian decision theory proposes that confidence is defined as the belief associated with the proposition that the observer has chosen or intends to choose. More precisely, confidence can be defined as the observer's belief that the chosen action maximizes utility [31]. Because organisms can make better decisions if they have a representation of the uncertainty and confidence associated with task-relevant variables [32], we observed that the confidence rating was modulated by SERT availability, both in the social and non-social learning tasks. Individuals showing high SERT availability were overconfident with respect to their probability of winning, especially concerning the worst option. This result reveals an effect of the serotoninergic system on a personal trait. The

modulation of the confidence rating could be accounted for by the fact that Individuals showing high SERT availability selected the strongest opponent less often, and therefore were less informed of the relative strength of the strongest opponents.

Individuals with high SERT availability were also more confident in their choices (regardless of social or non-social context) (**Figure S4B and S4C**) and exhibited higher reward-seeking traits (as assessed from BAS, **Figure S7**). Thus, high SERT individuals, who are more attracted to win in general, may seek to select inferior opponents (or the most rewarding slot machines), in agreement with the fact that they show a lower competitive index (**Figure 2D**). These findings suggest individuals with high SERT would presumably present a shorter half-life for released 5-HT at the synaptic cleft. This could facilitate confidence responses according to existing predispositions. These neurobehavioral findings could also explain why trait anxiety, presumably modulated by 5-HT, leads to differences in competitive confidence under stress [33].

Supplementary references

1. Mérida I, Reilhac A, Redouté J, Heckemann RA, Costes N, Hammers A. Multi-atlas attenuation correction supports full quantification of static and dynamic brain PET data in PET-MR. Physics in Medicine and Biology. 2017;62:2834–2858.
2. Reilhac A, Merida I, Irace Z, Stephenson MC, Weekes AA, Chen C, et al. Development of a dedicated rebinner with rigid motion correction for the mMR PET/MR Scanner, and Validation in a Large Cohort of 11C-PIB Scans. Journal of Nuclear Medicine. 2018;59:1761–1767.
3. Hammers A, Allom R, Koepp MJ, Free SL, Myers R, Lemieux L, et al. Three-dimensional maximum probability atlas of the human brain, with particular reference to the temporal lobe. Human Brain Mapping. 2003;19:224–247.
4. Lammertsma AA, Hume SP. Simplified reference tissue model for PET receptor studies. NeuroImage. 1996;4:153–158.
5. Kish SJ, Furukawa Y, Chang LJ, Tong J, Ginovart N, Wilson A, et al. Regional distribution of serotonin transporter protein in postmortem human brain: Is the cerebellum a SERT-free brain region? Nuclear Medicine and Biology. 2005;32:123–128.
6. Ashburner J. A fast diffeomorphic image registration algorithm. NeuroImage. 2007;38:95–113.
7. Ogden RT, Ojha A, Erlandsson K, Oquendo MA, Mann JJ, Parsey R v. In vivo quantification of serotonin transporters using [11C]DASB and positron emission tomography in humans: Modeling considerations. Journal of Cerebral Blood Flow and Metabolism. 2007;27:205–217.
8. Meyer JH, Houle S, Sagrati S, Carella A, Hussey DF, Ginovart N, et al. Brain serotonin transporter binding potential measured with carbon 11-labeled DASB positron emission tomography: Effects of major depressive episodes and severity of dysfunctional attitudes. Archives of General Psychiatry. 2004;61:1271–1279.
9. Praschak-Rieder N, Wilson AA, Hussey D, Carella A, Wei C, Ginovart N, et al. Effects of tryptophan depletion on the serotonin transporter in healthy humans. Biological Psychiatry. 2005;58:825–830.
10. Talbot PS, Frankle WG, Hwang DR, Huang Y, Suckow RF, Slifstein M, et al. Effects of reduced endogenous 5-HT on the in vivo binding of the serotonin transporter radioligand 11C-DASB in healthy humans. Synapse. 2005;55:164–175.
11. Carpenter LL, Anderson GM, Pelton GH, Gudin JA, Kirwin PDS, Price LH, et al. Tryptophan Depletion During Continuous CSF Sampling in Healthy Human Subjects. vol. 19. 1998.
12. Moreno FA, Parkinson D, Palmer C, Castro WL, Misiaszek J, el Khoury A, et al. CSF neurochemicals during tryptophan depletion in individuals with remitted depression and healthy controls. European Neuropsychopharmacology. 2010;20:18–24.
13. Paterson LM, Tyacke RJ, Nutt DJ, Knudsen GM. Measuring endogenous 5-HT release by emission tomography: Promises and pitfalls. Journal of Cerebral Blood Flow and Metabolism. 2010;30:1682–1706.
14. Rolls ET, Huang CC, Lin CP, Feng J, Joliot M. Automated anatomical labelling atlas 3. NeuroImage. 2020;206:116189.
15. Matthew Brett, Anton J-L, Valabregue R, Poline J-B. Region of interest analysis using an SPM toolbox [abstract]. Presented at the 8th International Conference on Functional

Mapping of the Human Brain, Sendai, Japan. p. Available on CD-ROM in NeuroImage, Vol 16, No 2.

16. Daunizeau J, Adam V, Rigoux L. VBA: A Probabilistic Treatment of Nonlinear Models for Neurobiological and Behavioural Data. PLoS Computational Biology. 2014;10.
17. Lenhard, W. & Lenhard, A. (2014). Hypothesis Tests for Comparing Correlations. available: https://www.psychometrica.de/correlation.html. Bibergau (Germany): Psychometrica. DOI: 10.13140/RG.2.1.2954.1367.
18. Kvaal K, Ulstein I, Nordhus IH, Engedal K. The Spielberger State-Trait Anxiety Inventory (STAI): The state scale in detecting mental disorders in geriatric patients. International Journal of Geriatric Psychiatry. 2005;20:629–634.
19. M. Becker, Morris H. DeGroot and JM. Measuring utility by a single-response sequential method. 1964:226–232.
20. Carver, Charles S., White TeriL (University of M. Behavioral Inhibition, Behavioral Activation, and Affective Responses to Impending Reward and Punishment: The BIS/BAS Scales. Journal of Pesonality and Social Psychology. 1994;67:319–333.
21. Rathus SA. Assertif Behavior Scale. 1973.
22. Pratto F, Sidanius J, Stallworth LM, Malle BF. Social Dominance Orientation: A Personality Variable Predicting Social and Political Attitudes. Journal of Personality and Social Psychology. 1994;67:741–763.
23. Lottem E, Banerjee D, Vertechi P, Sarra D, Lohuis MO, Mainen ZF. Activation of serotonin neurons promotes active persistence in a probabilistic foraging task. Nature Communications. 2018;9:1–12.
24. Eldar E, Roth C, Dayan P, Dolan RJ. Decodability of Reward Learning Signals Predicts Mood Fluctuations. Current Biology. 2018;28:1433-1439.e7.
25. Michely J, Eldar E, Martin IM, Dolan RJ. A mechanistic account of serotonin’s impact on mood. Nature Communications. 2020;11.
26. Michely J, Eldar E, Erdman A, Martin IM, Dolan RJ. SSRIs modulate asymmetric learning from reward and punishment. BioRxiv. 2020:2020.05.21.108266.
27. Bang D, Kishida KT, Lohrenz T, Tatter SB, Fleming SM, Montague PR, et al. Article Sub-second Dopamine and Serotonin Signaling in Human Striatum during Perceptual Decision-Making Sub-second Dopamine and Serotonin Signaling in Human Striatum during Perceptual Decision-Making. Neuron. 2020:1–12.
28. Moran RJ, Kishida KT, Lohrenz T, Saez I, Laxton AW, Witcher MR, et al. The Protective Action Encoding of Serotonin Transients in the Human Brain. Neuropsychopharmacology. 2018;43:1425–1435.
29. Kishida KT, Saez I, Lohrenz T, Witcher MR, Laxton AW, Tatter SB, et al. Subsecond dopamine fluctuations in human striatum encode superposed error signals about actual and counterfactual reward. Proceedings of the National Academy of Sciences of the United States of America. 2016;113:200–205.
30. Dayan P. Twenty-Five Lessons from Computational Neuromodulation. Neuron. 2012;76:240–256.
31. de Martino B, Fleming SM, Garrett N, Dolan RJ. Confidence in value-based choice. Nature Neuroscience. 2013;16:105–110.
32. Ma WJ, Jazayeri M. Neural coding of uncertainty and probability. Annual Review of Neuroscience. 2014;37:205–220.

33. Goette L, Bendahan S, Thoresen J, Hollis F, Sandi C. Stress pulls us apart: Anxiety leads to differences in competitive confidence under stress. Psychoneuroendocrinology. 2015;54:115–123.

Supplementary figure and table

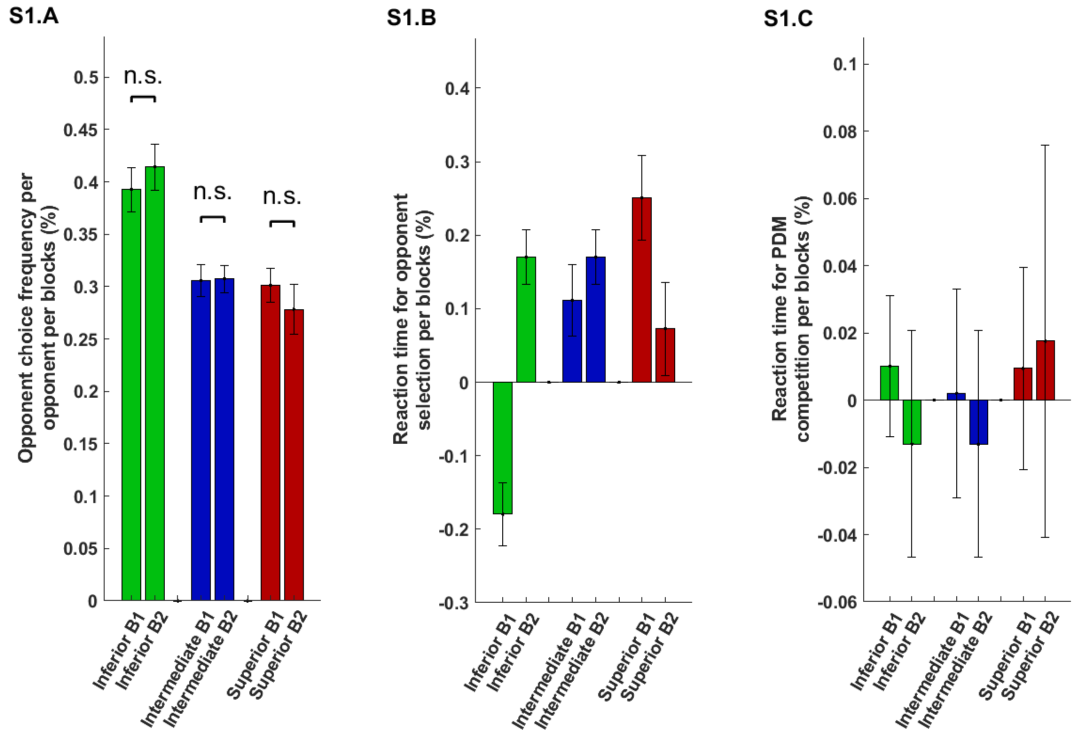

Figure S1: control for block effect in the social task. Results revealed significant interaction effect between the opponent and the block for the reaction time of the opponent selection ($F_{(2,58)}$=5.32; p=0.010) (**Figure S1.B**). No differences were observed for the frequency of choice nor for the reaction time at the PDM stage.

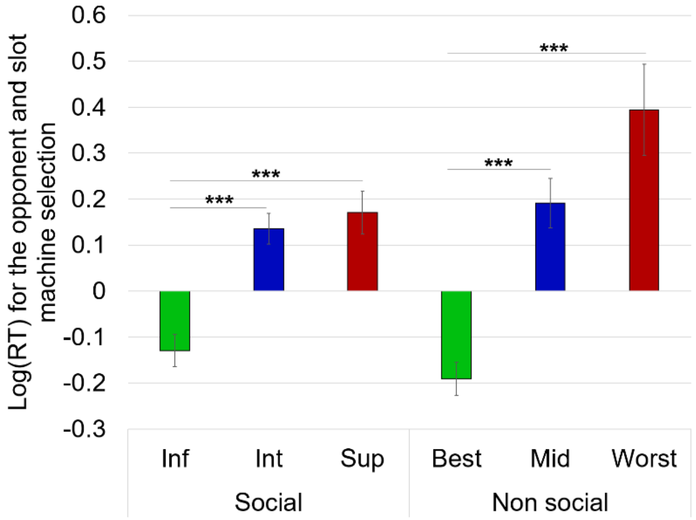

Figure S2: comparison of the reaction time for the opponent and slot machine selection. Results revealed a significant main effect of the opponent/slot machine ($F(2,58)=26.167$; $p<0.001$). Participants select the inferior opponent (or the best slot machine) more quickly than the intermediate or superior opponent (or middle and worst slot machine). There was also no main effect of the condition and no interaction effect between the condition and reward probabilities. ***$p<0.001$

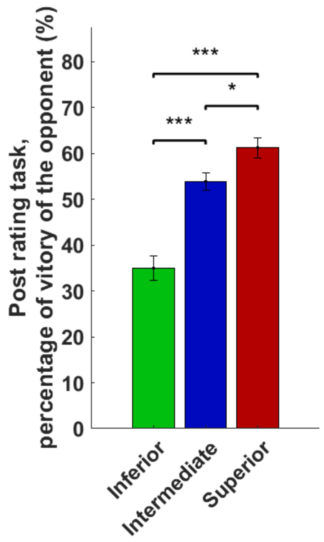

Figure S3: A. Post-ratings of the percentage of victories of the opponents (averaged across the two blocks of social task). Results of the repeated measures ANOVA revealed a significant difference in the evaluations ($F_{(2,58)}$=4.842, p=0.012). Participants rated the inferior opponents as having less victories (M=35.17 SEM=2.63) compared to the intermediate opponent (M=53.87 SEM=1.94) ($t_{(28)}$=-5.88, p<0.001) and the superior opponent (M=61.21 SEM=2.14) ($t_{(28)}$=-7.09, p<0.001). They also rated the intermediate opponents as having less victories compared to the superior opponent ($t_{(28)}$=-4.14, p=0.048). In addition, participant selected the best opponent among the pairs presented at the end of the task. Results revealed that participants were able to correctly choose the best opponent among the pair ($t_{(28)}$=17.96, p<0.001).

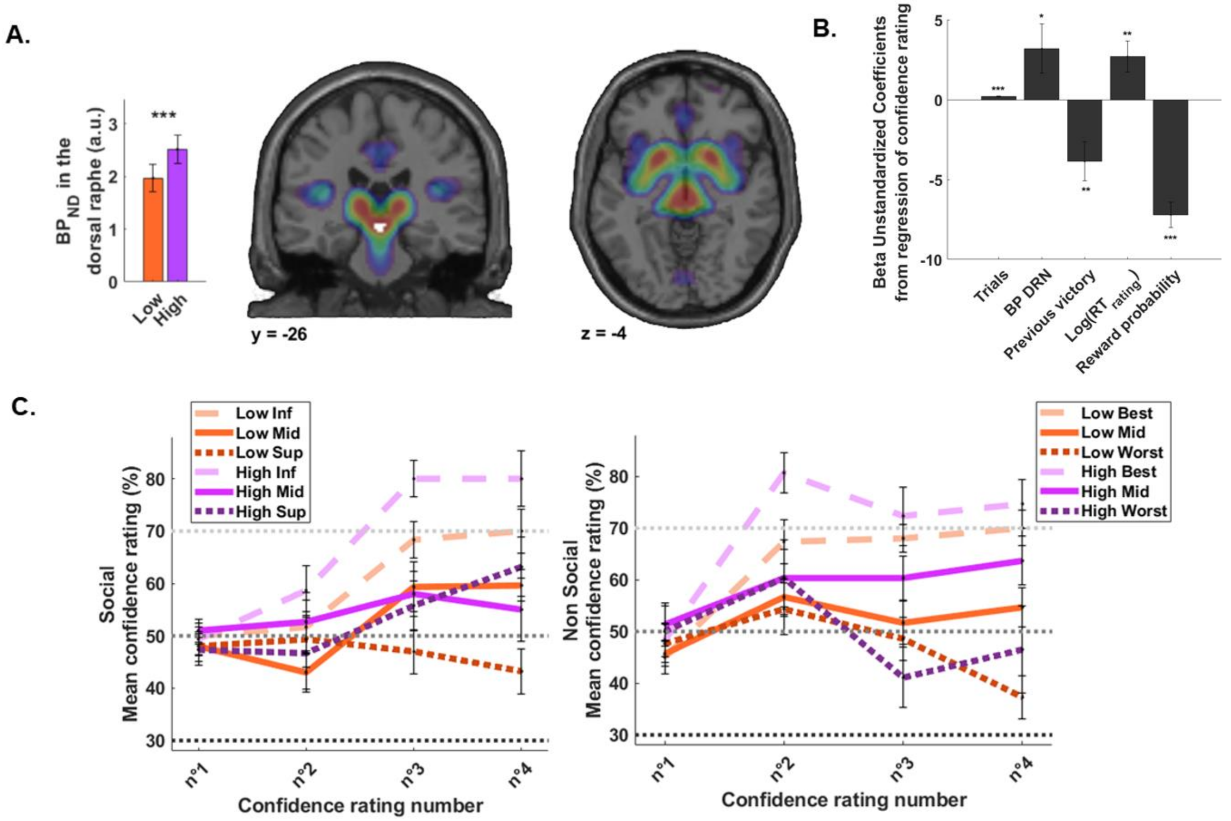

Figure S4. Link between Dorsal Raphe Nucleus Binding Potential and confidence rating

A. Average BP_{ND} brain map for the whole group of participants. On the left, the bar graph shows a median split of the participants based on their SERT BP_{ND} in the DRN. **B.** Beta coefficient resulting from the regression analysis of the confidence rating. The confidence rating is explained by the trial number, the reward probability, the previous outcomes at the trial t-1, the $\log(RT_{rating})$ of the rating, and the BP_{ND} in the DRN. **C**. Confidence ratings of the two groups were separated according to the median split for the social task (left side) and non-social task (right). Red lines indicate the confidence rating of the low BP_{ND} group, purple lines indicate the confidence rating of the high BP_{ND} group. Inf/Mid/Sup represent the opponent categories (with a probability of victory of 72% 50% and 28% respectively) and Best/ Mid/ Worst represent the slot machine categories (winning probability of 72% 50% and 28% respectively). Errors bars represent SEM. $*p<0.05$, $**p<0.05$, and $***p<0.001$. SERT=serotonin reuptake transporter, DRN=dorsal raphe nucleus

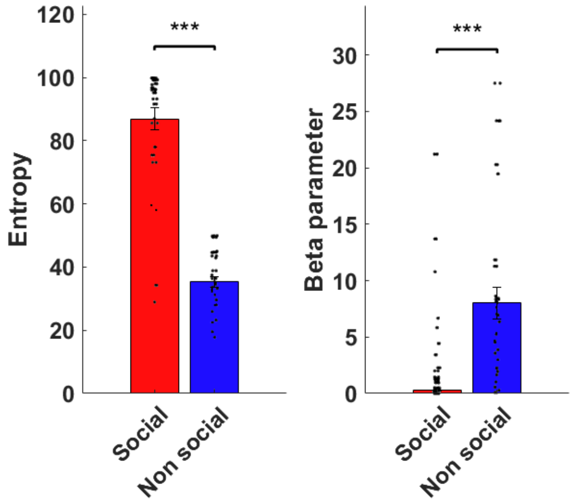

Figure S5: **Choice entropy and beta parameters comparison between social and non-social tasks**. On the left side the entropy of the choice is plotted. On the right side, the beta parameters estimated from the models are plotted. Both bar graph represents the mean value of the metric and dots represent the distribution of the participants. Entropy of the choice was calculated by subtracting Shannon entropy of the chosen option from the Shannon entropy of the unchosen option using the following formula: p*log(p) – (1-p) log(1-p).

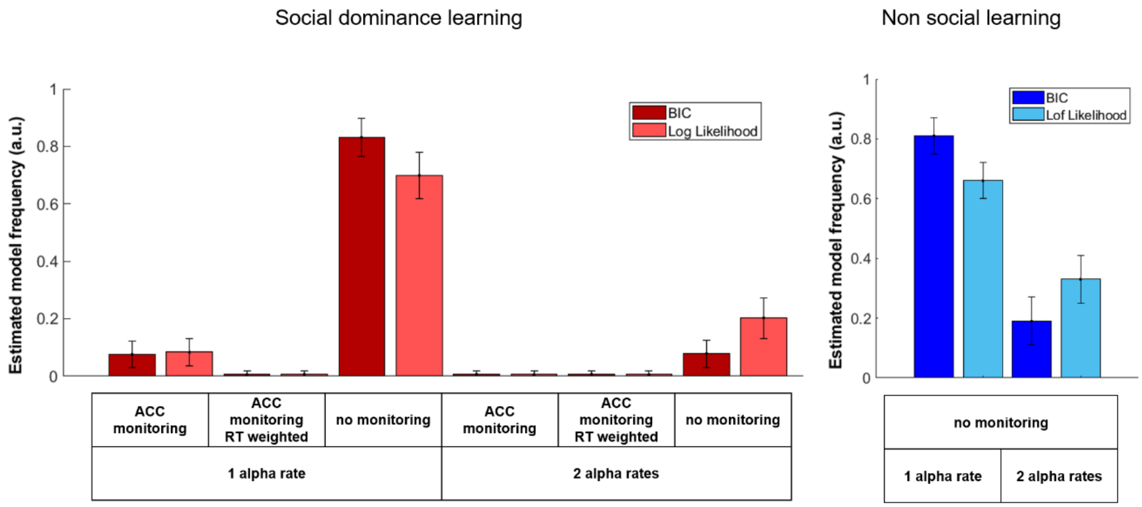

Figure S6. Bayesian model selection. On the left side, the estimated model frequency for the model set for the choices made during the social dominance hierarchy learning task. The model comparison indicated that the model with one alpha and no monitoring was the best describing the data. Light and dark bars represent the estimated frequency of each model in the population using both Bayesian Information Criterion (BIC) and Log likelihood (LL) as comparison metrics, respectively. Note that the model with the highest exceedance probability using the BIC criterion was used. On the right side the comparison for the model set for the non-social learning task is displayed (see also table S5 and supplemental experimental procedures).

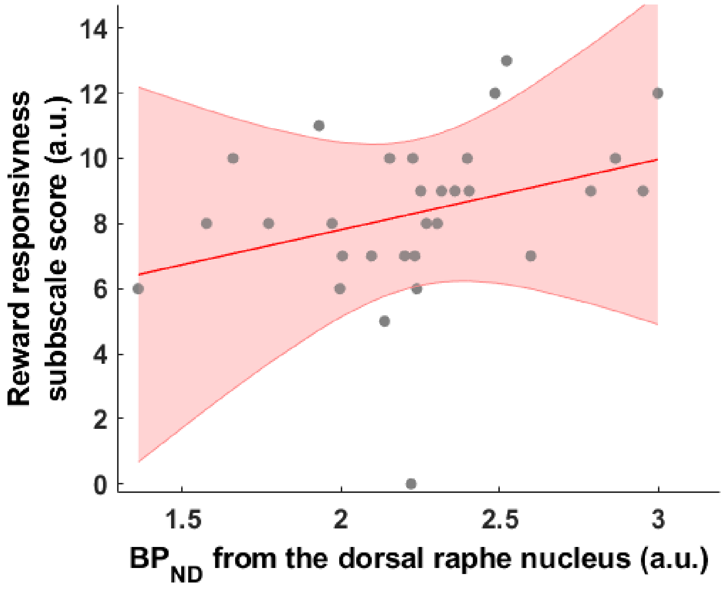

Figure S7: Positive correlation between the reward responsiveness subscale of the BAS scale and the SERT availability in the dorsal raphe nucleus ($p=0.019$, $r=0.427$). Results are consistent if the

participant who had a score of 0 at this subscale is removed ($p=0.019$, $r=0.433$). SERT=serotonin reuptake transporter.

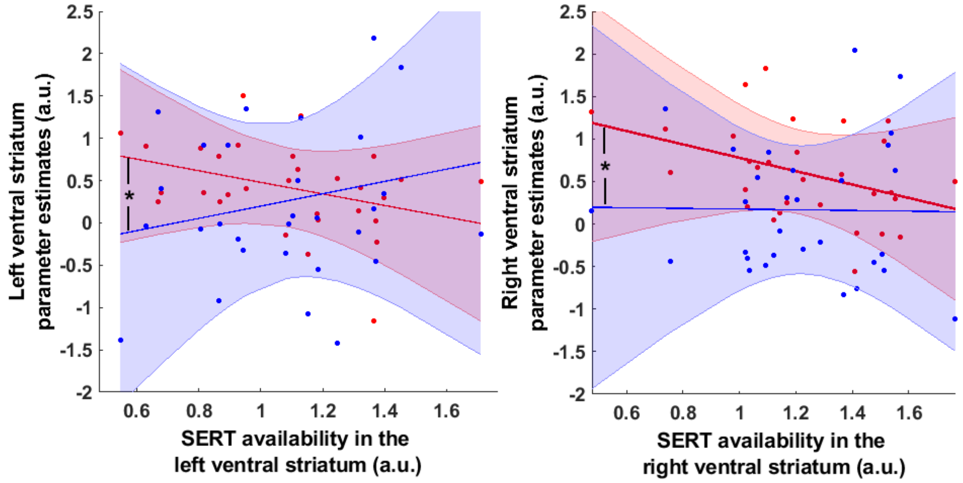

Figure S8. Correlation between the signal of social dominance status of the opponent and the SERT level in the ventral striatum. Slope difference between the social and non-social correlation estimated. Significant negative correlation between activity tracking the expected value of social victories SDS(t) and SERT availability in the ventral striatum (in red). No significant correlation between activity tracking the expected value of winning Q(t) and SERT availability in the ventral striatum (in blue). * Denotes significant differences between the slopes at a level of $p<0.05$, SERT=serotonin reuptake transporter level, SDS=social dominance status

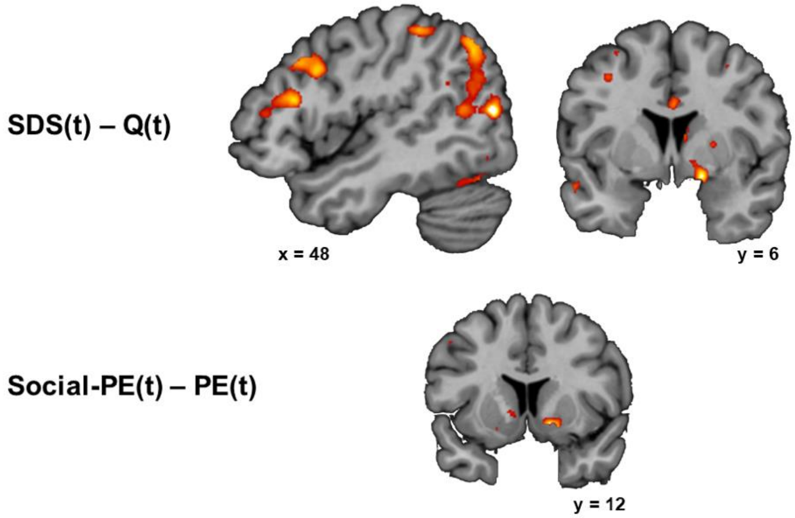

Figure S9: [Social > non-social] BOLD comparison. Direct comparison of the activity related to the SDS(t) and the Q(t) at the top, and comparison of the brain activity related to the SPE(t) and PE(t) at the bottom. Comparison of the expected value revealed that the activity of the dlPFC, the parietal lobule and the ventral part of the right putamen are significantly greater in the social condition compared to the non-social. Similar analysis revealed that the right putamen is more activated by the social PE(t) compare to the non-social PE(t). All statistical analyses were performed at a $p<0.05$ cluster level corrected for Family Wise Error at the whole brain level, with an initial cluster forming threshold of $p<0.001$ uncorrected. dlPFC=dorsolateral prefrontal cortex

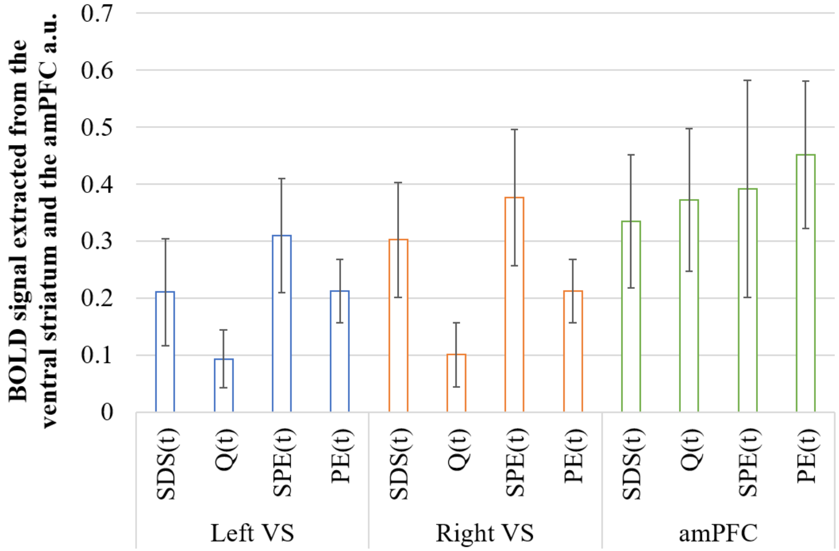

Figure S10: BOLD signal relative to the SDS/Q(t) and SPE/PE(t) for illustrative purpose. Extracted in the ventral striatum left (bleu) and right (orange) defined using the AAL3 atlas and in the amPFC (green), spherical ROI of 5mm radius and peak coordinates x:-4, y:52, z:-3 (adapted from Ligneul et al., 2016).

Table S1. Regions parametrically activated by the SDS(t+1). GLM1

**Region**	MNI coordinates **x**	**y**	**z**	**k**	**Z score**
Ventral striatum R	14	14	-8	4450	6.17
Ventral striatum L	-18	14	-9		5.5
anterior mPFC L	-4	57	-4	693	4.3
dlPFC R	50	32	18	416	4.3
Anterior cingulate cortex	-15	-46	38	863	4.49
Posterior cingulate cortex / precuneus	6	-46	48	424	3.96
Inferior temporal gyrus	-57	-56	-6	810	4.36
Angular gyrus R	40	-72	46	1287	4.31
Cerebellum	36	-74	-38	13068	5.74

Table S1. Regions parametrically varying with SDS(t+1). GLM1. All statistical analyses were performed at a $p<0.05$ cluster level corrected for Family Wise Error at the whole brain level, with an initial cluster forming threshold of $p<0.001$ uncorrected. Labeling of all regions was done using the peak activity and the AAL3 atlas.

Table S2. Regions parametrically activated by the SDS(t) and SPE(t). GLM2

	**Region**	**MNI coordinates**			**k**	**Z score**
		**x**	**y**	**z**		
SDS(t)	Putamen L	-16	14	-6	777	5.27
	Putamen R	16	14	-2	1552	4.7
	anterior mPFC	-4	52	-3	689	4.3
	Temporal middle gyrus R	57	-58	2	8656	5.19
SPE(t)	Caudate R	10	18	8	2768	5.93
	Caudate L	-9	14	2	2279	5.85
	Anterior mPFC	4	62	3	1638	4.66
	Superior frontal gyrus R	27	18	51	752	4.57
	Superior frontal gyrus L	-21	21	54	2508	5.74
	Middle cingulate gyrus L	-3	-39	33	4442	5.73
	Angular gyrus R	40	-60	28	4367	5.04
	Angular gyrus L	-44	-66	45	5980	6.09
	Inferior temporal gyrus L	-46	-51	-18	609	4.71
	Lingual gyrus R	12	-81	-9	12308	5.92

Table S2. Regions parametrically varying with SDS(t) and SPE(t). GLM2. All statistical analyses were performed at a $p<0.05$ cluster level corrected for Family Wise Error at the whole brain level, with an initial cluster forming threshold of $p<0.001$ uncorrected. Labeling of all regions was done using the peak activity and the AAL3 atlas.

Table S3. fMRI activity in the nonsocial task. GLM3 and GLM4

	**Region, hemisphere**	**MNI coordinates** x	y	z	**k**	**Z score**
Q(t+1) (GLM4)	anterior mPFC L	-6	50	-10	876	4.17
PE(t) (GLM5)	Caudate R	12	22	-4	1023	4.24
	Medial PFC gyrus L	-29	24	44	310	4.46
	Medial PFC gyrus L (2)	-9	51	-14	389	3.91
	Superior frontal gyrus R	9	66	0	395	4.24
	Medial posterior cingulate gyrus L	-8	-33	39	818	4.68
	Medial OFC gyrus L	-18	28	-16	338	4.65
	Superior temporal gyrus R	58	-16	2	447	4.41
Q(t) (GLM5)	anterior mPFC L	-2	39	-2	1053	4.71

Table S3. fMRI activity in the non-social task. GLM4 and GLM5. All statistical analyses were performed at a p<0.05 cluster level corrected for Family Wise Error at the whole brain level, with an initial cluster forming threshold of p<0.001 uncorrected otherwise noted. Labeling of all regions was done using the peak activity and the AAL3 atlas.

	Unstandardized Coefficients		Standardized Coefficients	t	Sig.	Collinearity Statistics	
	B	Std. Error	Beta			Tolerance	VIF
**(Constant)**	61.470	6.393		9.615	0.000		
**Trial**	0.193	0.027	0.225	7.089	0.000	0.899	1.112
**Reward Probability**	-7.209	0.799	-0.280	-9.018	0.000	0.933	1.072
**Previous Outcome**	-3.856	1.213	-0.101	-3.179	0.002	0.899	1.113
**RT_{rating}**	2.699	0.972	0.084	2.776	0.006	0.990	1.010
**$RT_{opponent}$**	-1.621	1.696	-0.030	-0.956	0.339	0.914	1.094
**BP DRN**	3.196	1.544	0.063	2.070	0.039	0.978	1.022
**Block**	-0.630	1.442	-0.015	-0.437	0.662	0.736	1.358
**Condition**	-0.803	1.438	-0.020	-0.558	0.577	0.708	1.412

Table S4: Mixed effect regression summary of the confidence rating.

The regression model includes the following predictors: Trial = trial number of the rating, Reward Probability = The reward probability of the chosen option (opponent or slot machine), the Previous Outcome = the results of the previous trial, RT_{rating} = the log(RT) of the confidence rating, $RT_{opponent}$ = the log(RT) of the choices (opponent/slot machine), BP DRN = the BP in the DRN, Block the number of block and Condition = the social vs non-social task. Unstandardized and Standardized beta coefficients for each variable are included in the linear model and their p-values associated (cf. sig). The tolerance metric allows verification that the multicollinearity assumption is not violated. If tolerance is around 0.1, it means that at least two explanatory variables are too collinear. The VIF, the variance inflation factor which measures the correlation and strength of correlation between the predictor variables in a regression model. A VIF score lower than 5 indicates that multicollinearity will not be a problem in the regression model.

		Description	BIC - sum	LL - sum	n param	Alpha		Beta	ω	Exceedance probability	
						win	loss			BIC	LL
**Social**	RL 1	1α - error update	-3259	-2958	2	0.37 ± 0.21		3.15 ± 5.42			
	RL 2	1α - 1ω error update	-3345	-2996	3	0.37 ± 0.19		2.60 ± 4.64	0.6 ± 0.19		
	RL 3	1α - no error update	-3232	-2931	1	0.39 ± 0.22		3.17 ± 5.03		0.99	0.99
	RL 4	2α - error update	-3300	-2951	3	0.39 ± 0.21	0.35 ± 0.24	3.29 ± 5.48			
	RL 5	2α - 1ω error update	-3393	-2996	4	0.39 ± 0.19	0.36 ± 0.22	2.85 ± 5.02	0.65 ± 0.18		
	RL 6	2α - no error update	-3284	-2936	2	0.38 ± 0.21	0.41 ± 0.25	3.68 ± 5.5			
**Non social**	RL 1	1α	-1482	-1236	2	0.27 ± 0.23		8.92 ± 9.7		0.85	0.7
	RL 2	2α	-1511	-1228	3	0.31 ± 0.2	0.30 ± 0.24	6.7 ± 5.05		0.15	0.3

Table S5. Model selection and parameters explaining the choices for the social and non-social learning tasks. Bayesian Information Criterion (BIC). Log Likelihood (LL). α=learning rate. β=inverse temperature. ω=sensitivity to response reaction time. See also the modeling section of the supplementary methods for the detail of the algorithm used for each model.

	Mean	Max	Min	STD	SEM
**Age**	23.5	32	19	2.8	0.5
**Body Mass Index**	22.07	33.00	0.00	2.64	1.33
**BECK**	7.33	33.00	0.00	7.67	1.33
**BAS** (total)	48.27	67.00	0.00	11.32	1.97
drive	10.00	14.00	0.00	2.44	0.42
fun seeking	6.97	12.00	0.00	2.43	0.42
reward *	8.33	13.00	0.00	2.48	0.43
**BIS** (total)	15.03	24.00	0.00	4.83	0.84
fillers	7.93	11.00	0.00	2.15	0.37
**STAI (total)**	94.57	108.00	81.00	5.98	1.04
trait	49.43	61.00	42.00	4.39	0.76
state	45.13	52.00	36.00	4.01	0.70
**Rathus**	98.57	112.00	74.00	8.48	1.48
**Social dominance orientation**	63.40	75.00	0.00	12.77	2.22

Table S6. Demographic summary and results of the correlation between personality traits and the Non-Displaceable Binding Potential of [^{11}C]-DASB in the dorsal raphe nucleus. Results revealed a significant positive correlation between the reward responsiveness subscale and the level of SERT available in the dorsal raphe nucleus (p=0.019, r=0.427), denoted by the Asterix (S6 Fig.). No other significant correlation was observed. To assess anxious temperament, they completed the Spielberger trait and state anxiety scale (Y-T and Y-S version). A distinction is made between the trait (YT), which is a general temperament, and the state (YS), which is more variable over time and corresponds to the person's current temperament. To measure depressive temperament, participants completed the BECK scale. Participants also completed the BIS-BAS questionnaire, which assesses two general motivational systems underlying behavior: the behavioral inhibition scale (BIS) and the behavioral approach system (BAS). Finally, a scale to assess the self-assertiveness and social orientation of individuals was also completed. Social assertiveness was assessed using the assertiveness questionnaire, and social orientation was assessed using the social dominance orientation scale.
